# Homogeneity and Possible Replacement of Populations of the Dengue Vectors *Aedes aegypti* and *Aedes albopictus* in Indonesia

**DOI:** 10.3389/fcimb.2021.705129

**Published:** 2021-07-07

**Authors:** Triwibowo Ambar Garjito, Widiarti Widiarti, Muhammad Choirul Hidajat, Sri Wahyuni Handayani, Mujiyono Mujiyono, Mega Tyas Prihatin, Rosichon Ubaidillah, Mohammad Sudomo, Tri Baskoro Tunggul Satoto, Sylvie Manguin, Laurent Gavotte, Roger Frutos

**Affiliations:** ^1^ Institute for Vector and Reservoir Control Research and Development, National Institute of Health Research and Development, The Ministry of Health of Indonesia, Salatiga, Indonesia; ^2^ Doctoral School of Medical Science, Faculty of Medicine, Diponegoro University, Semarang, Indonesia; ^3^ Research Center for Biology, Indonesian Institute of Sciences, Cibinong, Indonesia; ^4^ National Institute of Health Research and Development, The Ministry of Health of Indonesia, Jakarta, Indonesia; ^5^ Department of Parasitology, Faculty of Medicine, Public Health and Nursing, Gadjah Mada University, Yogyakarta, Indonesia; ^6^ HydroSciences Montpellier (UMR-HSM), IRD, CNRS, Montpellier, France; ^7^ Espace-Dev, University of Montpellier, Montpellier, France; ^8^ Cirad, UMR 17, Intertryp, Montpellier, France

**Keywords:** *Aedes aegypti*, *Aedes albopictus*, Indonesia, *cox*1, internal transcribed spacer 2 (ITS2)

## Abstract

Currently, *Aedes aegypti*, the principal vector of dengue virus in Indonesia, has spread throughout the archipelago. *Aedes albopictus* is also present. Invasion and high adaptability of the *Aedes* mosquitoes to all of these areas are closely related to their ecology and biology. Between June 2016 and July 2017, larval and adult mosquito collections were conducted in 43 locations in 25 provinces of Indonesia using standardized sampling methods for dengue vector surveillance. The samples collected were analyzed for polymorphism and phylogenetic relationship using the mitochondrial *cox*1 gene and the nuclear ribosomal internal transcribed spacer 2 (ITS2). Almost all *Ae. aegypti* samples collected in this study (89%) belonged to the same haplotype. A similar situation is observed with the nuclear ITS2 marker. Populations of *Ae. aegypti* characterized few years ago were genetically different. A closely related observation was made with *Aedes albopictus* for which the current populations are different from those described earlier. *Ae. aegypti* populations were found to be highly homogenous all over Indonesia with all samples belonging to the same maternal lineage. Although difficult to demonstrate formally, there is a possibility of population replacement. Although to a lower extent, a similar conclusion was reached with *Ae. albopictus.*

## Introduction

*Aedes aegypti* is known as a major vector of dengue viruses (family *Flaviviridae*, genus *Flavivirus*, DENV) (Simmons et al., 2012; Kraemer et al., 2015), yellow fever virus (family *Flaviviridae*, genus *Flavivirus*, YF) (da Costa-da-Silva et al., 2005; Kraemer et al., 2015; Yohan et al., 2018), zika virus (family *Flaviviridae*, genus *Flavivirus*, ZIKV) (Ja, 2016; Olson et al., 2020), and chikungunya virus (family *Togaviridae*, genus *Alphavirus*, CHIKV) (Kraemer et al., 2015; Yohan et al., 2018). This mosquito species originates from the forest of Africa and, since the 18th century, has spread *via* transcontinental trade throughout tropical and subtropical regions (Gubler, 1997; da Costa-da-Silva et al., 2005; Gubler, 2011; Joyce et al., 2018; Tedjou et al., 2019). In Southeast Asia, *Ae. aegypti* was formally identified for the first time in Malaysia and Thailand (1907) at the early 20th century (Urdaneta-Marquez and Failloux, 2011; Parimittr et al., 2018). *Ae. aegypti* was formally identified in Indonesia in 1908 (Leicester, 1908). A local strain of *Ae. aegypti*, the Medan strain, was first reported and successfully colonized in a laboratory in the 1930s (Kuno, 2010). Currently, the species is reported to have spread throughout the archipelago (Setiati et al., 2006; IVRCRD, 2018).

Another dengue vector species is the Asian tiger mosquito, *Aedes albopictus*. This species has for long been considered as a secondary vector of several viruses (Paupy et al., 2009; Grard et al., 2014; Goubert et al., 2016; Mulyatno et al., 2018). *Ae. albopictus* originates in the forests of Southeast Asia, and has spread worlwide since the 1970s (Moncayo et al., 2004). According to the Global invasive species database (http://www.issg.org/database/), this species has been recorded as one of the worst invasive species in the world. Currently, *Ae. albopictus* can be found in Asia, Africa, Europe, North and South America, and many locations in the Pacific and Indian oceans except Antartica (Paupy et al., 2009; Kraemer et al., 2015). As an invasive species, *Ae. albopictus* plays a potential role in triggering a re-emergence of arboviruses transmission in many locations. Recently, this species played an important role in Dengue, Chikungunya, and Zika outbreaks in both endemic and invaded regions (Rezza et al., 2007; Teixeira et al., 2009; Paupy et al., 2010; McKenzie et al., 2019; Lai et al., 2020).

The invasion and adaptation to all of these areas are closely related to their ecology and biology. *Ae. aegypti* has high adaptability to urban and peridomestic areas, where it breeds in the vicinity of human dwellings in a variety of artificial and natural containers in urban and rural areas (Kusriastuti and Sutomo, 2005; Kraemer et al., 2015; IVRCRD, 2018). This species is also recognized as the most anthropophilic mosquito and has the ability to blood-feed repeatedly on humans almost on a daily basis (Ritchie, 2014). This behavior may have contributed to the capacity of *Ae. aegypti* to cause high epidemics of dengue fever in Indonesia. A total of 68,407 dengue cases (incidence: 78.85/100,000) with 493 deaths (case fatality rates (CFR): 0.72%) were reported in 2017 (MoH Indonesia, 2018; Harapan et al., 2019). *Ae. albopictus* displays a strong ecological plasticity and has shown a remarkable capacity to adapt to urban and sub-urbans under various climate conditions, displacing *Ae. aegypti* population in some areas. *Ae. albopictus* has now become a significant vector of CHIKV and DENV (Knudsen., 1995; Paupy et al., 2009; Thiberville et al., 2013; Ngoagouni et al., 2015).

While an effective multivalent dengue vaccine is still under research and not yet available, vector control and entomological surveillance are the only reliable means of prevention and control of dengue fever (Wilder-Smith et al., 2010; Thisyakorn and Thisyakorn, 2014; Chang et al., 2015; Parra et al., 2018). Updated information on the genetic diversity and evolutionary patterns among *Ae. aegypti* and *Ae. albopictus* populations is needed to provide clues for better understanding the origin, the structuration, and the distribution of populations. Moreover, this is also a prerequisite to define differences in vector competence and capacity to transmit dengue virus, in ecological adaptations and in resistance to insecticides (Gupta and Preet, 2014; Yohan et al., 2018; Naim et al., 2020). However, a comprehensive information about genetic diversity and structuration of populations of *Ae. aegypti* and *Ae. albopictus* in Indonesia is still missing.

Therefore, we investigated the genetic diversity, evolutionary relationship, and distribution of *Ae. aegypti* and *Ae. albopictus* mosquitoes collected in different locations and islands, from Western Sumatra (Aceh) to Eastern Indonesia (Papua) using the mitochondrial *cox*1 or COI gene and the internal transcribed spacer 2 (ITS2) of the ribosomal DNA as target sequences.

## Material and Methods

### Collection and Rearing of Mosquitoes

Larva, pupa, and adult mosquitoes were collected using standardized sampling methods for dengue vector surveillance (Focks and Special Program for Research and Training in Tropical Diseases (TDR), 2003; Kusriastuti and Sutomo, 2005; WHO, 2011; Ritchie, 2014; WHO, 2016; MoH Indonesia, 2018; Harapan et al., 2019). In each house, larvae and pupae from different containers were put in different plastic bags. All samples were then transported to a field laboratory. Larvae and pupae were reared in the field laboratory for 3 days until the emergence of adult mosquitoes. Mosquitoes were then morphologically identified, sorted according to locality, and preserved in 250 µl of RNAlater (Ambion-Thermo Fisher Scientific, Watham, USA). This large sampling campaign was conducted as part of a nationwide program supervised by the Ministry of Health. Mosquitoes were collected during this campaign as a cohort of samples for use in different projects. This explains why the samples were stored individually in RNAlater even though no virus detection was conducted in this work. They were then stored at −80°C until further analysis. Larvae which did not emerge after 3 days were preserved the same way as adult mosquito samples for further analysis. All mosquito samples were individual samples, and only female mosquitoes were used as samples.

### DNA Extraction, Amplification and Sequencing

Whole DNA from each mosquito was individually extracted using a DNeasy^®^ Blood & Tissue Kit (Qiagen, Hilden, Germany) according to the manufacturer’s standard protocol. ITS2 and *cox*1 (COI) were selected as target sequence because they are the most used sequences for phylogenetic and population structure analyses of mosquitoes. They are present in databases. They were also used in previous works on the genotyping of *Aedes* mosquitoes in Indonesia, allowing thus for comparison. The amplification of *cox*1 was conducted using the primers CI-N-2087 (5′-AAT TTC GGT CAG TTA ATA ATA TAG-3′ and TY-J-1460 (5′-TAC AAT TTA TCG CCT AAA CTT CAG CC-3′) as previously described (Rueanghiran, et al., 2011; Ngoagouni et al., 2015). The ITS2 sequence was amplified using the primers ITS2a (5′-TGT GAA CTG CAG GAC ACA T-3′) and ITS2b (5′-TAT GCT TAA ATT CAG GGG GT-3′). PCR reactions were carried out using the GoTaq^®^ Green Master Mix (Promega, Madison, WI, USA). The conditions for PCR amplification of the *cox*1 gene were as follows: one cycle at 94°C for 1 min for initial denaturation, followed by five cycles at 94°C for 30 s, 45°C for 40 s, and 72°C for 1 min. This was then followed by 35 cycles at 94°C for 30 s, 44°C for 40 s, and 72°C for 1 min, and by a final extension step at 72°C for 10 min (Ngoagouni et al., 2015). PCR thermocycling conditions for ITS2 were as follows: 94°C for 10 min; followed by 40 cycles of denaturation at 94°C for 1 min, annealing at 56°C for 45 s, and elongation at 72°C for 1 min; followed by a final extension step at 72°C for 10 min. PCR products were electrophoresed in 1.5% agarose gel and visualized by SYBR^®^ safe DNA gel stain (Invitrogen, Carlsbad, CA, USA) using a Biorad Molecular Image Gel Doc XR (Biorad Laboratories Inc, California, USA). A 100-bp DNA ladder was used for calculating the size of the PCR products. Amplicons were purified using Applied Biosystems ExoSAP-IT™ (Thermo Fisher Scientific, Vilnius, Lithuania). Cycle sequencing was performed using the primers listed above and an Applied Biosystems BigDye™ Terminator v.3.1 Cycle Sequencing Kit (Life Technologies Cooperation, Austin, TX, USA). To remove unincorporated BigDye^®^ terminators and salts, cycle sequencing products were purified using a BigDye^®^ Xterminator Purification Kit (Life technologies, Bedford, MA, USA). Sequence data were obtained using a DNA sequencer (Applied Biosystems^®^ 3500 Genetic Analyzer) and analyzed using the Sequencing Analysis 6 program (Applied Biosystems). All sequences have been deposited in Genbank under the accession numbers MW280620 to MW280792, MW280794 to MW280797 and MW280800 to MW280818 for *Ae. aegypti cox*1 sequences. The accession numbers for *Ae. aegypti* ITS2 sequences range from MW288143 to MW288145 and MW290431 to MW290468. The accession numbers for *Ae. albopictus cox*1 sequences are MW280793, MW280798, MW280799, and MW283303 to MW283318. The accession numbers for *Ae. albopictus* ITS2 sequences are MW287155 to MW287157. The accession numbers of the *cox*1 and ITS2 sequences of the unknown species *Aedes* sp (sls25_Asp) are MW286812 and MW293720, respectively.

### Polymorphism and Phylogenetic Analysis

Sequences were analyzed for definition of haplotypes using DnaSP software v.6.12.03 (Rozas et al., 2017). The relationship between haplotypes, based on pairwise difference to generate a minimum spanning tree (MST) and minimum spanning network (MSN), was calculated and modeled using Network software and Hapstar v. 0.7. Multiple alignment and phylogenetic analysis were performed using the SaeView package (Gouy et al., 2010). Phylogenetic trees were built using maximum-likelihood (ML) with the general time reversible model with gama distributed with four discrete categories (GTR + I + G). The clade support was assessed *via* 500 bootstrap replicates.

## Results

### Mosquito Collection

**Figure 1:**
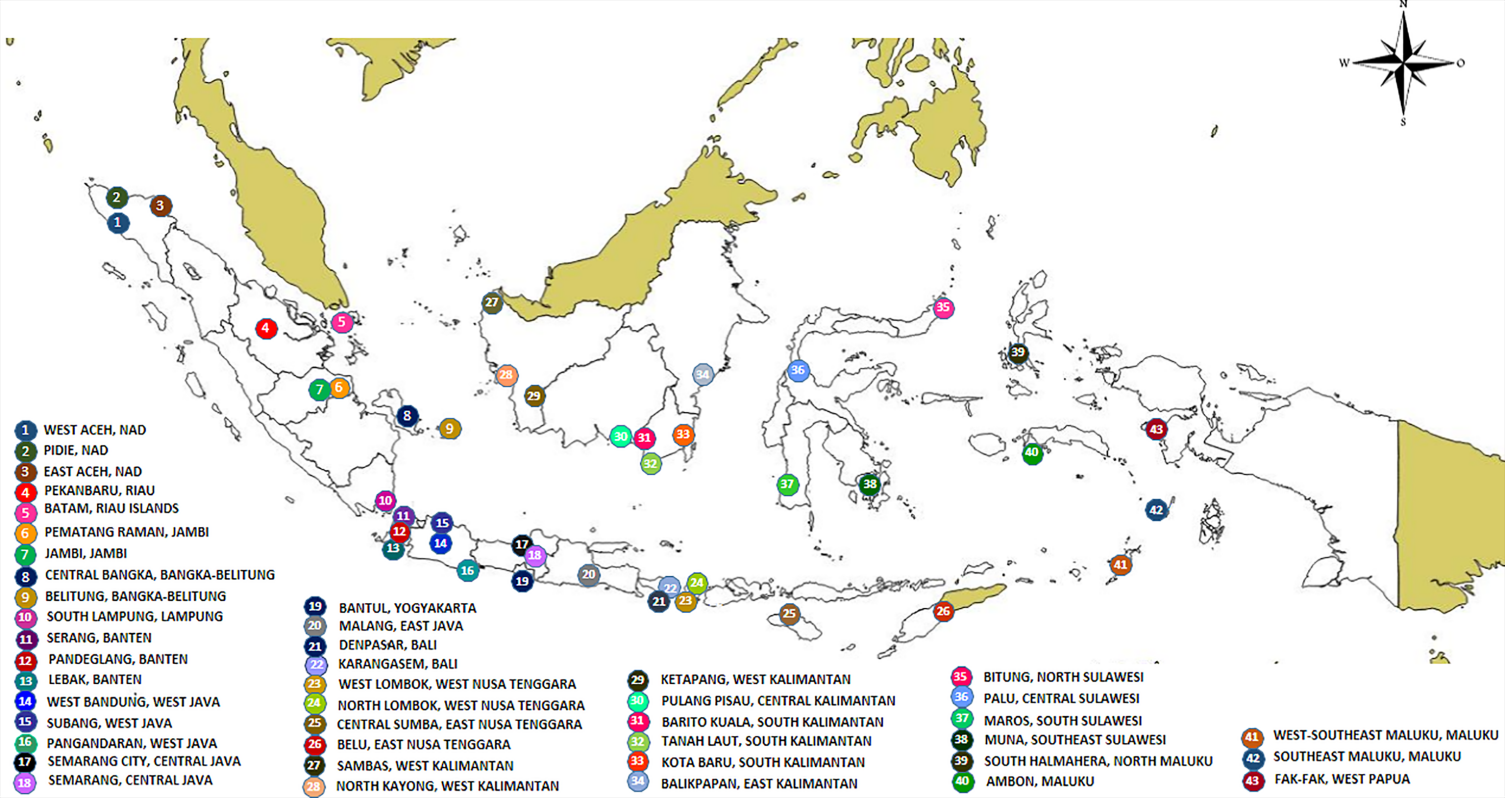
Map of sampling sites.

Collections were conducted in 43 districts/municipalities in 25 dengue-endemic provinces in Indonesia (**Supplementary Table 1** and **Figure 1**). These provinces were Aceh, Riau, Riau Islands, Jambi, Bangka-Belitung, Lampung, Banten, West Java, Central Java, Yogyakarta, East Java, West Kalimantan, South Kalimantan, Central Kalimantan, East Kalimantan, Bali, West Nusa Tenggara, East Nusa Tenggara, North Sulawesi, Central Sulawesi, South Sulawesi, Southeast Sulawesi, Maluku, North Maluku, and West Papua. Sampling of larva and adult mosquitoes was conducted as part of the 2nd year of the “Rikhus Vektora” project in July–August 2016 in 28 locations, the WHO project SEINO (#1611945) in September–October 2016 in six locations, and subsequently in nine locations as part of the 3rd year of “Rikhus Vektora” project in May–July 2017 (**Figure 1**). A total of 60,873 *Ae. aegypti* mosquitoes were collected: 2,184 adults, 54,251 larvae, and 4,438 adults obtained from reared larvae. With respect to *Ae. albopictus*, 16,223 mosquitoes were collected with the following breakdown: 4,957 adults, 9,638 larvae, and 1,628 adults obtained from reared larvae. From these samples 196 *Ae. aegypti* mosquitoes were sequenced: 34 adults and 162 adults obtained from reared larvae. For *Ae. albopictus*, 19 samples were sequenced, two adults and 17 adults obtained from reared larvae.

### Phylogenetic Relationships of the Collected Samples

The samples collected in this work fell into three different branches, both for *cox*1 and ITS2 (**Supplementary Figure 1**). These branches corresponded to *Ae. aegypti*, *Ae. albopictus*, and another undetermined *Aedes* species. The latter sample was therefore named sls25_Asp (**Supplementary Figure 1**). The *cox*1 gene phylogeny showed the presence of two main clusters in *Ae. aegypti*, Cluster Aae1 and Cluster Aae2, with Cluster Aae1 being separated into two subclusters: Subcluster Aae1a and Subcluster Aae1b (**Supplementary Table 1** and **Supplementary Figure 1**). These clusters were separated by very low bootstrap values indicating that the tree was not well structured and that the samples belonged to the same population (**Supplementary Figure 1**). However, with the *cox*1 gene from *Ae. albopictus*, three clusters could be identified with strong bootstraps (100), Cluster Aal1, Cluster Aal2, and Cluster Aal3 (**Supplementary Table 2** and **Supplementary Figure 1**).

### Presence of a Yet Unidentified Species

The sample sls25_Asp from Maros in South Sulawesi (site No 36 in **Figure 1**) was initially misidentified in the field as *Culex quinquefasciatus*. This sample was branching apart from *Ae. aegypti* and *Ae. albopictus* for the *cox*1 gene indicating that it was neither *Ae. aegypti* nor *Ae. albopictus* (**Supplementary Figure 1**). It was also different from *Cx. quinquefasciatus* which was used as outgroup. The phylogeny of the ITS2 sequences showed a similar result. The sample sls25_Asp was different from *Ae. aegypti* and *Ae. albopictus* (**Supplementary Figure 2**). The ITS2 sequence showed a best hit with an *Aedes polynesiensis* mosquito from Fidji (AY822662) with a percentage of identity of 88.24%, whereas the *cox*1 sequence displayed a best hit with an *Ae. albopictus* sample from Vietnam (HQ398902) with 91.95% identity. However, there was no *cox*1 sequence for *Ae. polynesiensis* in Genbank, and it was thus impossible to confirm if the *cox*1 gene would also link sls25_Asp to *Ae. polynesiensis.* A morphological analysis showed that the closest species, although still with morphological differences, was *Aedes paullusi* (data not shown). There is no *cox*1 or ITS2 records for *Ae. paullusi* in databases. The *Ae. albopictus* and *Ae. polynesiensis* hits for sls25_Asp *cox*1 and ITS2 sequences, respectively, might simply be default hits due of the lack of relevant sequences in the databases.

### Phylogeny and Polymorphism of *Aedes aegypti cox*1 Gene

**Figure 2:**
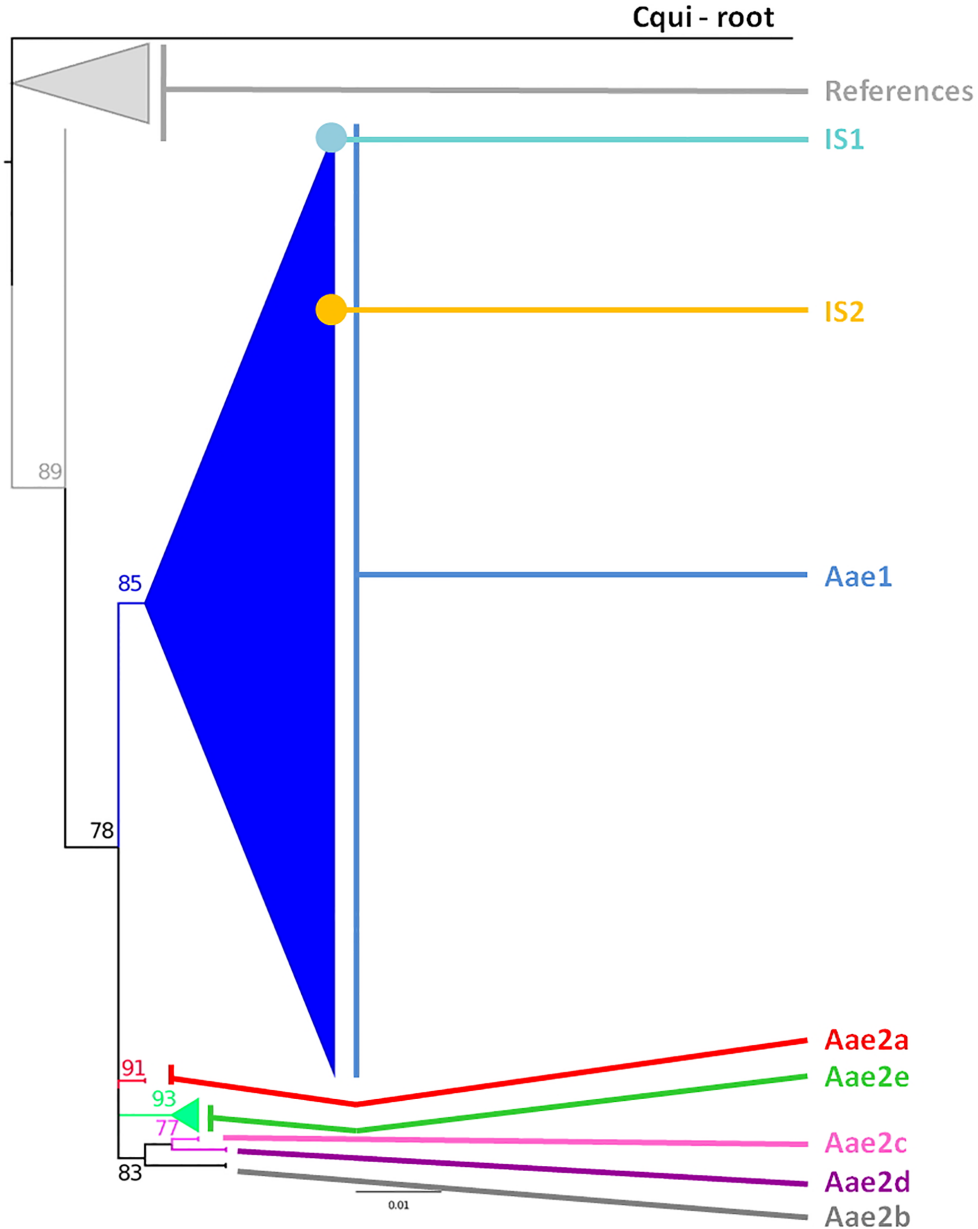
Phylogeny of the *Aedes aegypti cox*1 gene. The phylogenetic trees were built using maximum-likelihood (ML) with the general time reversible model with gama distributed with four discrete categories (GTR + I + G). The clade support was assessed *via* 500 bootstrap replicates. The tree was rooted using the *Culex quinquefasciatus cox*1 gene (MK265737) as outgroup. The color code is that of the *cox*1 subclusters shown in **Supplementary Table 1**: light gray: References, dark blue: Subcluster Aae1, red: Subcluster Aae2a, dark gray: Subcluster Aae2b, pink: Subcluster Aae2c, purple: Subcluster Aae2d, green: Subcluster Aae2e, light blue: individual sample 1, yellow: individual sample 2, black: root. “References” correspond to the *Ae. aegypti cox*1 sequences published by Yohan et al. (2018) from samples collected in 2013 whose accession numbers are KP334259 to KP334269 and KP869121 to KP89126.

**Figure 3:**
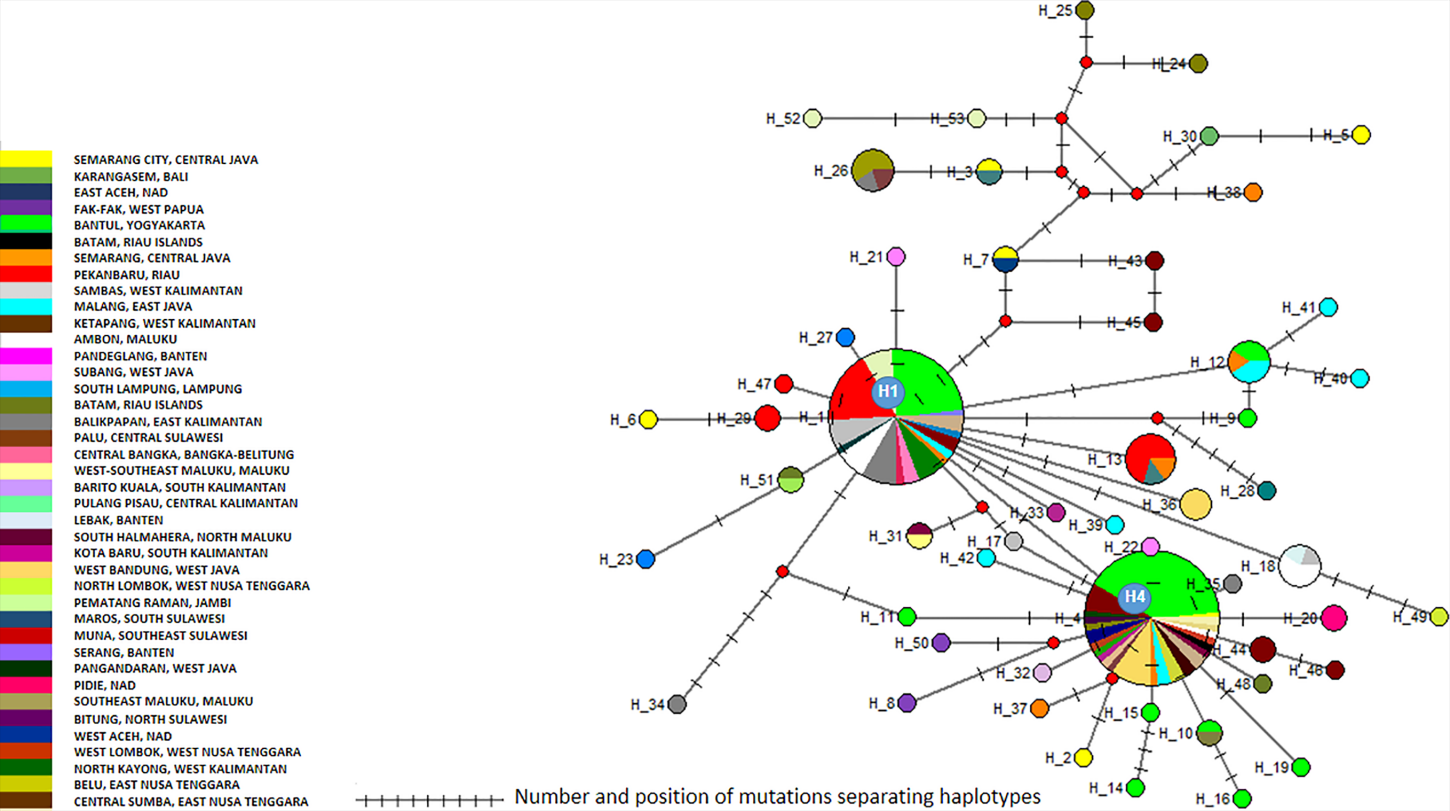
Network of *Aedes aegypti cox*1 haplotypes. The figure represents the frequency of each haplotype of the *cox*1 gene of *Ae. aegypti* in the regions sampled. The network represents the number of mutations between the haplotypes and their location.

The *cox*1 sequences from 196 samples collected for this work were compared to the only other source of *Ae. aegypti cox*1 sequences from Indonesia, a 17-sample collection from 2013 in the North Coast of Central Java (Yohan et al., 2018). These samples, identified in the tree by their accession numbers from KP869121 to KP869126 and KP334259 to KP334269, make a completely separate cluster (**Figure 2**). The genetic distance between the sequences from this work and those reported by Yohan et al. (2018) ranged from 0.4 to 3.1%. With a mutation rate of the *cox*1 gene in insects ranging from 2.4% Mya^−1^ to 3.5% Mya^−1^ (Brower, 1994; Papadopoulou et al., 2010), the time needed for the accumulation of these mutations ranges from 114,285 to 167,000 years for a mutation rate of 2.4% Mya^−1^ and from 885,714 to 1,291,667 years for a mutation rate of 3.1% Mya^−1^. When blasting the *cox*1 sequences from this work against Genbank data, the sequences from previously *Ae. aegypti* identified from Indonesia in 2013 (Yohan et al., 2018) did not respond as best hits. However, best hits were obtained with the same score with a series of nine *cox*1 sequences of *Ae. aegypti* mosquitoes captured in Peru, Cambodia, Puerto Rico, India, Georgia, England, and Germany (**Supplementary Table 3**). Subcluster Aae2a showed two best hits both from Kenya (**Supplementary Table 3**). Subclusters Aae2b and Aae2d had only one corresponding best hit in Genbank from Mozambique and Haiti, respectively (**Supplementary Table 4**). Subcluster Aae2c showed five best hits with the same score from Egypt and Kenya (**Supplementary Table 3**). Interestingly, Subcluster Aae2e displayed four best hits with the same score, but only one was a wild-type mosquito captured in Haiti. The other three best hits corresponded to the reference strains reared in laboratory conditions of Liverpool and RED (**Supplementary Table 3**). Finally, each of the two individual samples diverging from Cluster 1, 46_Aae (IS1) and 28-1-Aae (IS2) displayed a different best hit. IS1 showed a best hit with a mosquito collected in Russia, whereas IS2 showed a best hit with *Aedes aegypti formosus*, which is considered an ancestral feral population from sub-Saharan Africa (Powell and Tabachnick, 2013; Gloria-Soria et al., 2016) (**Supplementary Table 3**). The breakdown into individual haplotypes showed that Cluster Aae1 comprised 176 samples out of 198 (89%) and 39 haplotypes out of 53 (73.6%) with two of them, H1 and H4, being the most represented (**Supplementary Tables 1**, **3** and **Figure 3**). H1 haplotype comprised 57 samples (32.85%) whereas the haplotype H4 contained 61 samples (34.6%) (**Supplementary Table 1**). Subcluster Aae1a comprised 16 haplotypes, including haplotype H1, and 75 samples, whereas Subcluster Aae1b contained haplotype H4 and 22 other haplotypes for a total of 102 samples (**Supplementary Table 1**).

### Phylogeographic Distribution of *Aedes aegypti cox*1 Lineages

Cluster Aae1 was, as expected, present everywhere with the exception of East Aceh and North Lombok (**Supplementary Figure 3** and **Supplementary Table 1**). No correlation could be found between any cluster and any location. When considering the geographic distribution of the haplotypes, a lack of correlation was also observed (**Supplementary Figure 4**). Only a default correlation could be observed, *i.e.* rare haplotypes from a region with few samples. However, this is a sampling bias and is not significant.

### Phylogeny and Polymorphism of *Aedes aegypti* ITS2

**Figure 4:**
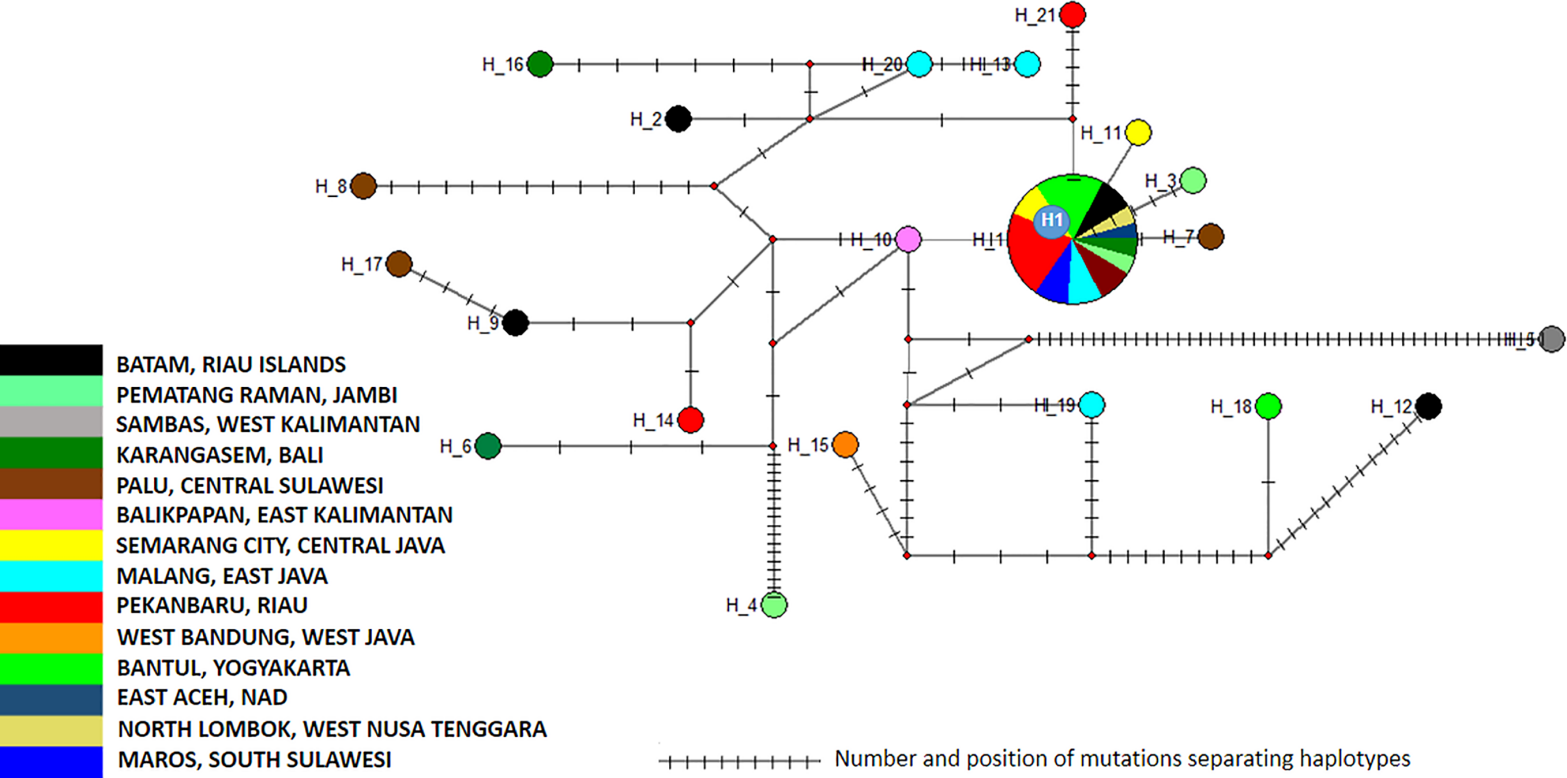
Network of *Aedes aegypti* ITS2 sequences. The figure represents the frequency of each haplotype of the ITS2 sequence of *Ae. aegypti* in the regions sampled. The network represents the number of mutations between the haplotypes and their location.

**Figure 5:**
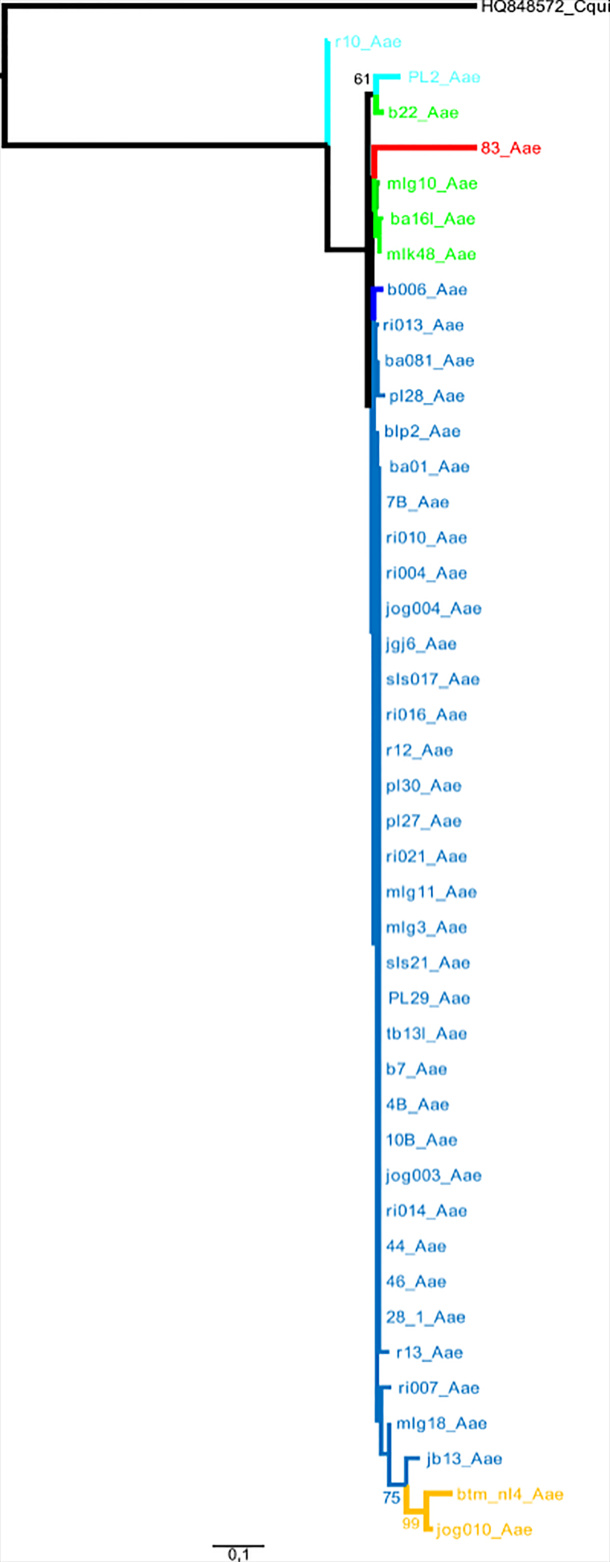
Phylogeny of the *Aedes aegypti* ITS2 sequences. The phylogenetic tree was built using maximum-likelihood (ML) with the general time reversible model with gama distributed with four discrete categories (GTR + I + G). The clade support was assessed *via* 500 bootstrap replicates. The tree was rooted using the *Culex quinquefasciatus* ITS2 sequence (HQ848572) as outgroup. The color code used is that of the ITS2 subclusters given in **Supplementary Table 1**.

The 40 sequences were distributed into two clusters and 21 haplotypes (**Supplementary Table 1**, **Figure 4** and **Supplementary Table 5**). Cluster 1 is divided into four subclusters (1a to 1d), which displayed limited variations (**Figure 5**). As a consequence, Cluster 1 gathered 42 samples and 23 haplotypes representing all the sequence variations observed within this monophyletic group (**Figure 5**). The different haplotypes were closely related with a maximum relative distance of 11.54% (**Supplementary Table 5**). Cluster 2 comprised only four samples, each one corresponding to a different haplotype. They were very closely related with an overall variation of 0.51% (**Supplementary Table 5**).

### Phylogeographic Distribution of *Aedes aegypti* ITS2 Sequences

The analysis of the polymorphism of *Ae. aegypti* ITS2 haplotype in this study showed that cluster 1 was the dominant one (**Supplementary Figure 5**). Subcluster 1a displayed the most extensive distribution, covering Sumatra (East Aceh-Aceh, Pematang Raman-Jambi, Pekan Baru-Riau, South Lampung-Lampung), Java (West Bandung-West Java, Semarang-Central Java, Bantul-Yogyakarta, Malang-East Java), Kalimantan (Sambas-West Kalimantan, Balikpapan-East Kalimantan), Bali (Karangasem), West Nusa Tenggara (Lombok), and Sulawesi (Palu-Central Sulawesi, Maros-South Sulawesi) (**Supplementary Figure 5**). Subcluster 1a showed a best hit with mosquitoes collected in Russia, Sri Lanka. The other subclusters, namely 1b, 1c, and 1d, showed a limited distribution. Subcluster 1b was only found in the Sambas-West Kalimantan region, while Subcluster 1c was identified in two locations: Batam-Riau Islands and Bantul-Yogyakarta. Subcluster 1d was found in two locations, *i.e.* Pematang Raman-Jambi and Palu-Central Sulawesi (**Supplementary Figure 5** and **Supplementary Table 5**). Cluster 2 had a more limited distribution. This cluster was found in Karangasem-Bali, Ambon-Maluku, Malang-East Java and Batam-Riau islands (**Supplementary Figure 5** and **Supplementary Table 5**).

### Phylogeographic Distribution, Phylogeny and Polymorphism of *Aedes albopictus cox*1 and ITS2

**Figure 6:**
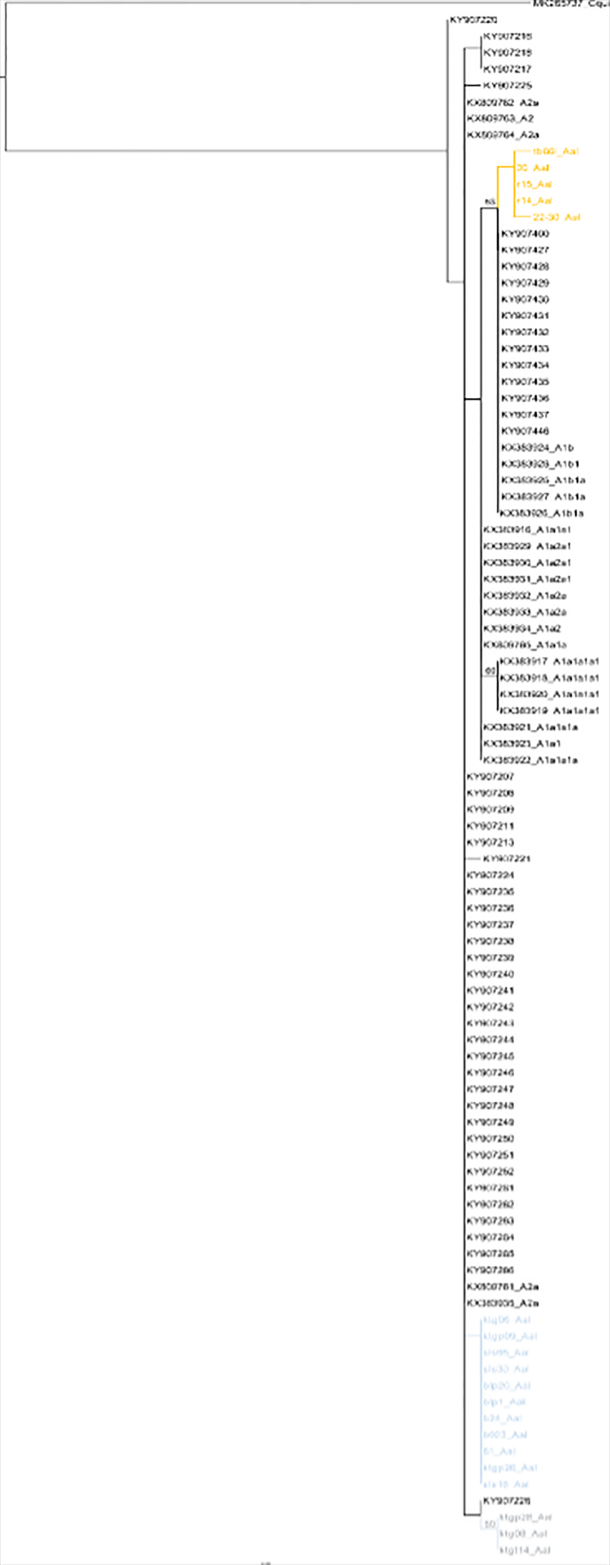
Phylogeny of *Aedes albopictus cox*1 genes. The phylogenetic tree was built using maximum-likelihood (ML) with the general time reversible model with gama distributed with four discrete categories (GTR + I + G). The clade support was assessed *via* 500 bootstrap replicates. The tree was rooted using the *Culex quinquefasciatus cox*1 gene (MK265737) as outgroup. The color code used is that of the *cox*1 subclusters displayed in **Supplementary Table 2**. Gray: Subcluster Aal1, green: Subcluster Aal2, orange: Subcluster Aal3.

**Figure 7:**
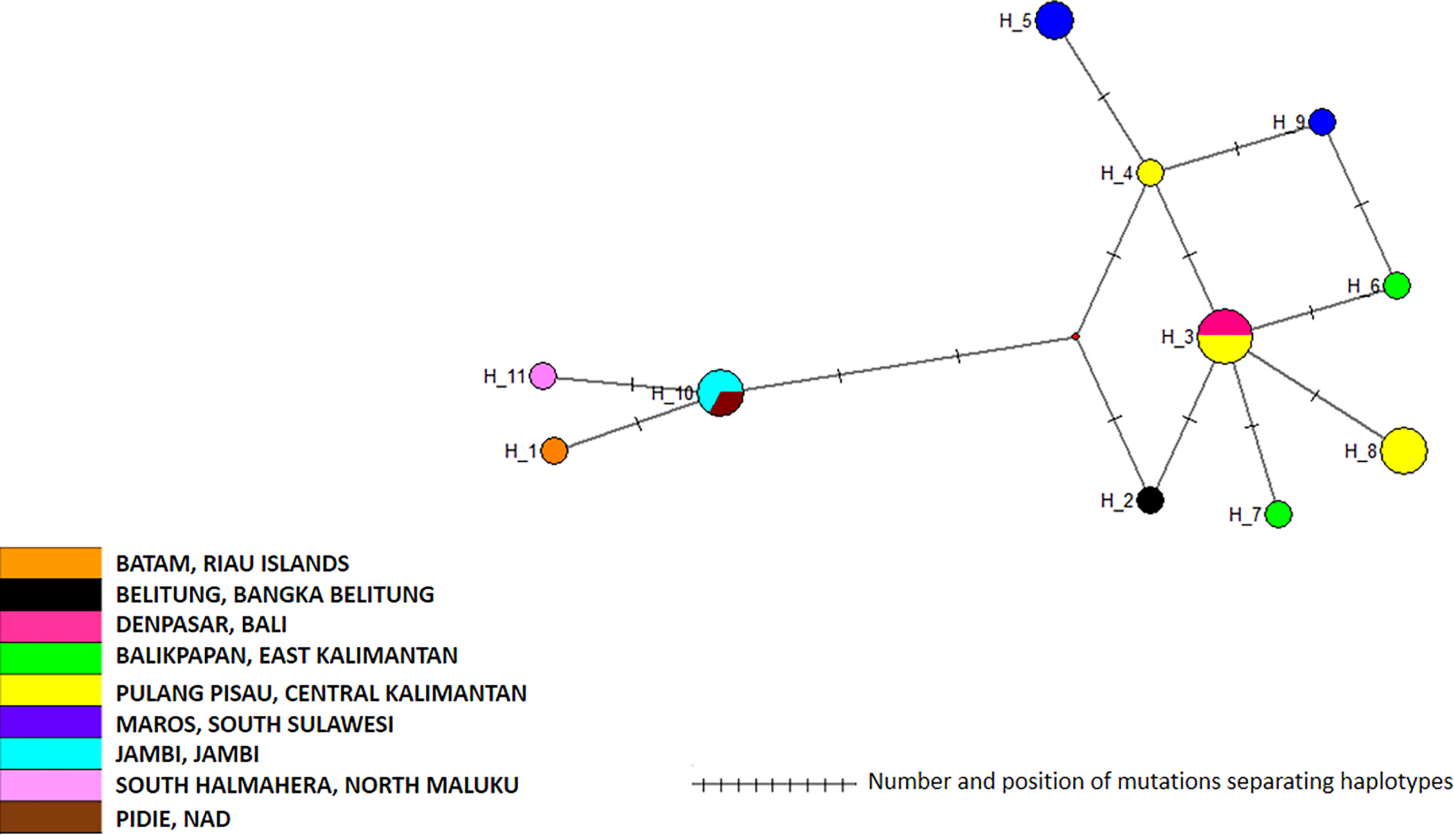
Network of *Aedes albopictus cox*1 haplotypes. The figure represents the frequency of each haplotype of the *cox*1 gene of *Ae. albopictus* in the regions sampled. The network represents the number of mutations between the haplotypes and their location on the gene sequence.

The 19 *cox*1 sequences of the *Ae. albopictus* samples collected in this work were compared to those released by Maynard et al. (2017) who collected samples in Jakarta in 2012, in Waingapu (Sumba) in 2013, and in Timika (Papua) in 2015. Sequences were also compared to those released by Battaglia et al. (2016) who established a worldwide classification of *Ae. albopictus cox*1 haplogroups (Battaglia et al., 2016). The COI sequences from Maynard et al. (2017) matched perfectly the haplogroups defined by Battaglia et al. (2016) and were distributed within two different haplogroups, A2a and A1b1a. The sequences obtained in this work did not correspond to the sequences reported by Maynard et al. (2017) and did not match any of the haplogroups defined by Battaglia et al. (2016) (**Figure 6**). Out of the three clusters identified within the *Ae. albopictus* sequences reported in this work, Cluster Aal1 was closer, although clearly different, to the haplogroups A2a; Cluster Aal3 was closer, although different, to the haplogroup A1ba1; and Cluster Aal2 was not close to any haplogroup. A total of 11 different haplotypes were found (**Figure 7** and **Supplementary Table 2**). The genetic distance between the *cox*1 sequences reported in this work and those from Maynard et al. (2017) ranges from 0.4 to 1.3%, depending on the sample. The time needed to accumulate the number of mutations separating the samples from this work to those described by Maynard et al. (2017) ranges from 114,285 to 167,000 years for a mutation rate of 2.4% Mya^−1^ and from 371,428 to 542,000 years for a mutation rate of 3.5% Mya^−1^. Cluster Aal1 was found only in Central Kalimantan, whereas the other two clusters were spread over different provinces (**Supplementary Figure 6**). However, the sample size is too small to draw any significant conclusion on the phylogeography. When blasted on databases, Cluster Aal1 and Cluster Aal2 both displayed best hits with the same *Ae. albopictus* populations from The Philippines but with differing percentage of identity ranging from 99.51 to 99.85% (**Supplementary Table 4**). Cluster Aal3 showed best hits with invasive populations of *Ae. albopictus* found in D.R. Congo, China, Thailand, Greece, Brazil, and the USA with 99.65 to 99.84% of identity (**Supplementary Table 4**). With respect to ITS2, the number of sequences available (only three) was too small to draw a conclusion. Nevertheless, they corresponded to invasive populations of *Ae. albopictus* found in Italy, Georgia, Israel, or Sri Lanka (**Supplementary Table 6**).

## Discussion

Dengue, which is the fastest spreading arbovirus disease worldwide, is also the first ranking vector-borne disease in Indonesia and, thus, a national health priority. In the absence of treatment and commercially available vaccine, vector management remains the only way to control the disease. However, in order to do so, the knowledge of vector population structure is an obligate prerequisite. The capacity of vectoring a given pathogen is not correlated with the species but instead with the population. Some populations of *Ae. aegypti* or *Ae. albopictus* are more prone to disseminate a given pathogen than others (Alto et al., 2008; Reinhold et al., 2018). Therefore, a species should be regarded as a metapopulation or the combination of cross-fertile genetically distinct populations displaying differing phenotypic traits (Garjito et al., 2020). Vector competence is one of these phenotypic traits, which in *Aedes* and other mosquitoes, was shown to be related to specific populations (Beerntsen et al., 2000; Severson and Behura, 2016) and not to the species *per se*. Deciphering the structure of the vector populations is thus essential.

Although dengue is the transmissible disease ranking number one in Indonesia, there have been very limited works on the analysis of the structure of the populations of *Aedes* with only one study on *Ae. Aegypti* (Yohan et al., 2018) and one on *Ae. albopictus* (Maynard et al., 2017). In both cases, the number of sampling sites was very limited. To these must be added studies aiming at assessing the stability of populations in the framework of a *Wolbachia* release program in Yogyakarta (Rašić et al., 2015). However, in this case, the genetic diversity was assessed with microsatellites and SNPs only on a sample from the city of Yogyakarta in South Central Java (Rašić et al., 2015). This work is, to our knowledge, the first one exploring the genetic diversity of *Ae. aegypti* and *Ae. albopictus* throughout Indonesia, a huge archipelago of more than 13,000 islands spanning 5,271 km from east to west and 2,210 km from north to south.

A first conclusion from this work is the homogeneity of the *Ae. aegypti* populations found all over Indonesia. With respect to the *cox*1 sequences, all *Ae. aegypti* samples belonged to the same maternal lineage. Variations were observed and clusters were described, but they simply represent a polymorphism within a monophyletic population. All clusters identified correspond to co-circulating variants. The main difference is that one cluster, Cluster Aae2, comprised samples displaying a larger polymorphism. Cluster Aae1, and in particular the haplotypes H1 and H4, seemed to be populations with a very high colonizing and demographic potential. These two haplotypes represent each about 30% of the samples collected all over Indonesia. They represent indeed the very same population with very limited or no polymorphism at all with Cluster Aae1 making up 87% of all samples and being present everywhere in Indonesia. Considering the size and structure of Indonesia, an archipelago spanning from the Indian Ocean to the Pacific Ocean, this is unexpected. One would have instead expected patched populations differing from one island to the other. What is observed is exactly the contrary, the same population throughout the whole country.

Owing to the mutation rate of the *cox*1 gene in *Ae. aegypti* (Brower, 1994; Papadopoulou et al., 2010), the population described in this work cannot have evolved from the populations previously described. Even in the case of introgression, the maternal lineage remains the same. What is described in this case is a different maternal lineage. This indicates that the observed population is allogenic. The lack of BLAST best hits with the previously described Indonesian populations and the occurrence of BLAST best hits with populations from other parts of the world also support this allogenic origin. Two hypotheses must then be considered to explain the massive presence of this homogeneous and allogenic population of *Ae. aegypti*. The first hypothesis is that the population described in this work has been completely missed in previous studies. Previous sampling conducted in 2013 yielded genetically different populations (Yohan et al., 2018) but were very limited and covered a limited zone (Yohan et al., 2018). This result could then be considered a consequence of a sampling bias, all mosquitoes from the previous study (Yohan et al., 2018) having been captured in a specific area, *i.e.* Northern Coastal Central Java. However, considering the extension of Cluster Aae1 throughout Indonesia and its overwhelming presence among samples (87%), it seems unlikely that it would have been missed in the 2013 sampling campaign. Furthermore, Cluster Aae1 was found to be strongly present in this same Northern Coastal Central Java area. The second hypothesis is that this allogenic population might have invaded Indonesia after the 2013 sampling. This invasion would have been very fast then, within a maximum of three years between 2013, date of the former sampling, and 2016, date of our first sampling. However, owing to the very limited information available on the previous populations, it is not possible to formally demonstrate this replacement hypothesis. Nevertheless, the ITS2 marker showed a similar trend; and considering the high potential of nuclear DNA for recombination and variation and finding the same ITS2 cluster all over Indonesia confirm the presence of a set of genetically closely related populations in Indonesia with one specific population characterized by two very closely related, monophyletic maternal haplotypes, H1 and H4. These haplotypes seem to be highly invasive most likely due to higher demographic and adaptability potentials. This also suggests that assortative mating might occur, which restricts greatly intraspecies breeding with preexisting populations.

*Ae. aegypti* has been shown to be highly susceptible to satyrization by *Ae. albopictus* leading to the replacement of *Ae. aegypti* populations by *Ae. albopictus* ones, thus explaining in part the invasive potential of the latter (Bargielowski and Lounibos, 2016). However, what is observed in Indonesia does not match this model despite the presence of populations of *Ae. albopictus* described as invasive in other parts of the world. What is seen in Indonesia is instead a homogeneous population of *Ae. aegypti* occupying all the archipelago and outcompeting *Ae. albopictus*. As per *Ae. aegypti*, the populations of *Ae. albopictus* collected in Indonesia do not correspond to those previously described from 2012 to 2015 (Maynard et al., 2017). Unlike populations described by Maynard et al. (2017), they also do not correspond to the haplogroups designed on samples from 2013 (Battaglia et al., 2016). They are related but not the same. Owing to the rate of mutation of the *cox*1 gene in insects, 2.4% Mya^−1^ to 3.5% Mya^−1^ depending on the model (Brower, 1994; Papadopoulou et al., 2010), the variations observed are not compatible with an evolution of previous local populations and indicate an invasion by allogenic populations. However, just as per *Ae. aegypti* the very limited number of previous studies makes it difficult to formally demonstrate a replacement.

The domestication of *Ae. aegypti* and *Ae. albopictus* is a process closely linked to the development of the human society and in particular to long distance mobility, transportation of goods, and international trade (Paupy et al., 2009). The current expansion of the *Aedes*-borne diseases is by far a consequence of the global economy. *Ae. aegypti* and *Ae. albopictus*, like all living organisms, are structured in metapopulations, which differ slightly one from the other and due to massive international transportation are distributed all over the world within the areas suitable for the survival of these species. The mobility of these populations from one place to another is a stochastic event, which depends on the place of departure, the place of arrival, the genetic and physiological traits of the populations involved, the economic situation, and the commercial exchanges and routes at a given time. This is to our knowledge the first report of such a massive homogeneity of a population of *Ae. aegypti* over such a large area.

One limitation of this work is the lack of previous data at the scale of the whole country. Only two studies have been conducted prior to our work on the genotyping of *Aedes* mosquitoes in Indonesia. There is only one report on the genotyping of *Ae. aegypti* but only on few samples and only in one restricted area in northern coastal central Java (Yohan et al., 2018). This lack of previous data prevents us from fully analyzing and concluding on the putative replacement of populations that the phylogenetic analyses suggest. Regarding the analysis of *Ae. albopictus*, the same problem occurred with only one previous study (Maynard et al., 2017) thus preventing the conduct of a fully significant comparison. On another hand, a major limitation of this work is the limited number of *Ae. albopictus* samples and sequences of ITS2. This is due to a limitation in funding which prevented the conduct of a full scale sequencing program. Further analyses of *Ae. albopictus* diversity in Indonesia should be conducted to complete the current study.

## Conclusions

A general consequence of our results is that populations are changing over time, even throughout a very large archipelago. Whatever the population, established or invasive, *Ae. aegypti* and *Ae. albopictus* mosquitoes will have to breed in the human environment. Then, the best way to prevent any population of vector from thriving is certainly to implement vector control at a very local level, at maximum at the community level, essentially by eliminating breeding sites using very simple and affordable means of control such as containers and garbage removal. The strategy of prevention of dengue transmission through community participation currently recommended in Indonesia will most likely be the most successful of all. This approach named 3M for “Menutup” for covering water containers, “Menguras” for cleaning water containers, and “Mengubur” for burying discarded containers, is implemented under the responsibility of families in each household with at least one person in charge of monitoring *Aedes* larvae in all water storage (Paupy et al., 2009; MoH Indonesia, 2015). This strategy shed light on what is most needed for the successful control of *Aedes*-borne diseases, not big science, big management or big strategies but simply information, education, people awareness and community-based management.

## Data Availability Statement

The datasets presented in this study can be found in online repositories. The names of the repository/repositories and accession number(s) can be found in the article/**Supplementary Material**.

## Ethics Statement

Specific permissions were not required for field *Aedes* larval collections. Oral consent to inspect the *Aedes* breeding places in the household was obtained from the homeowners and local government authorities. Formal approval to conduct these activities was granted by the Ethical Commission Board of the NIHRD, Ministry of Health, Indonesia (No. LB.02.01/5.2/KE.003/2016, January 13, 2016; and No. LB.02.01/5.2/KE.020/2017, February 6, 2017).

## Author Contributions

Conceptualization: TG, W, MH, RU, MS, and TS. Data curation: TG, SH, M, and MP. Investigation: TG, SH, M, and MP. Formal analysis: TG, MP, MH, RF, and LG. Resources: TG, MP, M, MH, and SH. Methodology: TG, RF, and LG. Project administration: MH and TG. Software: RF, LG, and TG. Supervision: SM and RF. Validation: RF, SM, TG, and LG. Writing—original draft: TG and RF. Writing—review and editing: RF, SM, and TG. All authors contributed to the article and approved the submitted version.

## Funding

The research was supported by the Institute for Vector and Reservoir Control Research and Development, National Institute of Health Research and Development, Ministry of Health Indonesia under the project Rikhus Vektora 2016–2017 and as part of the WHO project SEINO1611945 in 2016. TG, RF, SM, and TS were supported in part by the PHC Nusantara projects Zika & Co and SOCIAL, co-funded by the French Ministry of Foreign Affairs and RISTEKDIKTI under the Indonesian Ministry of Education and Culture.

## Conflict of Interest

The authors declare that the research was conducted in the absence of any commercial or financial relationships that could be construed as a potential conflict of interest.

## Supplementary Material

Supplementary Figure 1
*cox*1 gene phylogeny of the collected samples. The phylogenetic trees were built using maximum-likelihood (ML) with the general time reversible model with gama distributed with four discrete categories (GTR + I + G). The clade support was assessed *via* 500 bootstrap replicates. The tree was rooted using the *Culex quinquefasciatus cox*1 gene (MK265737) as outgroup.

Supplementary Figure 2 ITS2 phylogeny of the collected samples. The phylogenetic tree was built using maximum-likelihood (ML) with the general time reversible model with gama distributed with four discrete categories (GTR + I + G). The clade support was assessed *via* 500 bootstrap replicates. The tree was rooted using the *Culex quinquefasciatus* ITS2 sequence (HQ848572) as outgroup. The color code used is that of the ITS2 clusters of *Ae. aegypti* and *Ae. albopictus* displayed in **Supplementary Tables 1** and **2**, respectively. The color code for *Ae. aegypti* ITS2 clusters is: blue: Cluster 1a, red: Cluster 1b, orange: Cluster 1c.

Supplementary Figure 3 Geographic distribution of *Aedes aegypti cox*1 clusters.

Supplementary Figure 4 Geographic distribution of *Aedes aegypti cox*1 haplotypes.

Supplementary Figure 5 Geographic distribution of *Aedes aegypti* ITS2 sequences.

Supplementary Figure 6 Geographic distribution of *Aedes albopictus cox*1 haplotypes.

Supplementary Table 1 Specimens and sampling localities of *Aedes aegypti.*

Supplementary Table 2 Specimens and sampling localities of *Aedes albopictus*.

Supplementary Table 3 Polymorphism of *Aedes aegypti cox*1 haplotypes from Indonesia.

Supplementary Table 4 Polymorphism of *Aedes albopictus cox*1 haplotypes from Indonesia.

Supplementary Table 5 Polymorphism of *Aedes aegypti* ITS2 haplotypes from Indonesia.

Supplementary Table 6 Polymorphism of *Aedes albopictus* ITS2 haplotypes from Indonesia.
