# Supplementary material for: Homogeneity and Possible Replacement of Populations of the Dengue Vectors Aedes aegypti and Aedes albopictus in Indonesia
Source: Front Cell Infect Microbiol. 2021 Jul 7;11:705129. doi: 10.3389/fcimb.2021.705129 (PMC8294392; doi:10.3389/fcimb.2021.705129)
Supplement: Supplementary Figure 1 — cox1 gene phylogeny of the collected samples. The phylogenetic trees were built using maximum-likelihood (ML) with the general time reversible model with gama distributed with four discrete categories (GTR + I + G). The clade support was assessed via 500 bootstrap replicates. The tree was rooted using the Culex quinquefasciatus cox1 gene (MK265737) as outgroup. [file DataSheet_1.zip › Presentation 4.PPTX]

## Slide 1
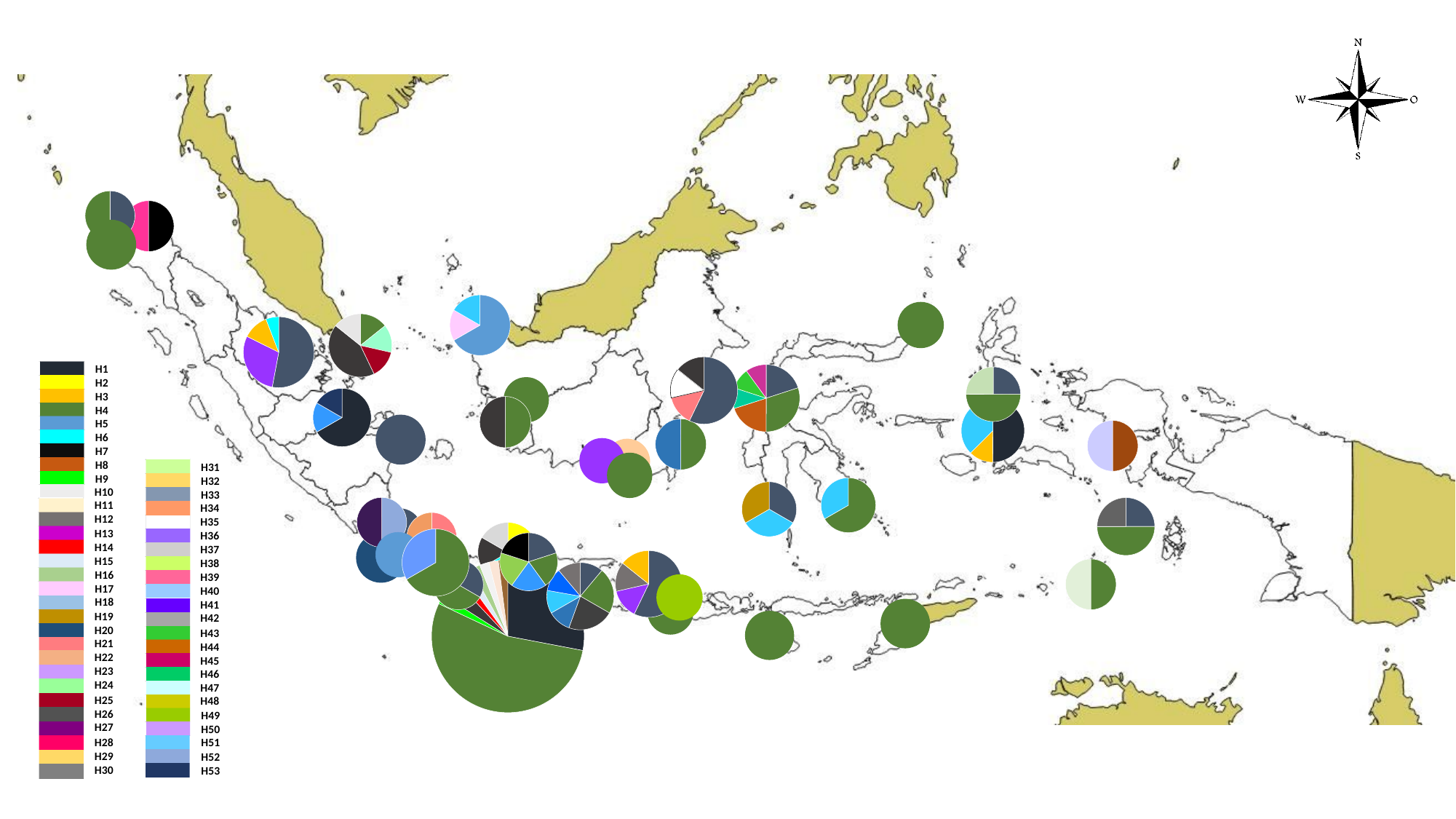

### Chart
| Category | Sales |
|---|---|
| haplotype 1 | 1.0 |
| haplotype 2 | 0.0 |
| haplotype 3 | 0.0 |
| haplotype 4 | 1.0 |
| haplotype 5 | 0.0 |
| haplotype 6 | 0.0 |
| haplotype 7 | 0.0 |
| haplotype 8 | 0.0 |
### Chart
| Category | Sales |
|---|---|
| haplotype 1 | 0.0 |
| haplotype 2 | 0.0 |
| haplotype 3 | 0.0 |
| haplotype 4 | 0.0 |
| haplotype 5 | 0.0 |
| haplotype 6 | 1.0 |
| haplotype 7 | 0.0 |
| haplotype 8 | 1.0 |
### Chart
| Category | Sales |
|---|---|
| haplotype 1 | 1.0 |
| haplotype 2 | 0.0 |
| haplotype 3 | 0.0 |
| haplotype 4 | 0.0 |
| haplotype 5 | 0.0 |
| haplotype 6 | 0.0 |
| haplotype 7 | 0.0 |
| haplotype 8 | 0.0 |
### Chart
| Category | Sales |
|---|---|
| haplotype 1 | 4.0 |
| haplotype 2 | 0.0 |
| haplotype 3 | 0.0 |
| haplotype 4 | 0.0 |
| haplotype 5 | 0.0 |
| haplotype 6 | 0.0 |
| haplotype 7 | 0.0 |
| haplotype 8 | 0.0 |
| h17 | 1.0 |
| h18 | 1.0 |
### Chart
| Category | Sales |
|---|---|
| haplotype 1 | 1.0 |
| haplotype 2 | 0.0 |
| haplotype 3 | 0.0 |
| haplotype 4 | 0.0 |
| haplotype 5 | 0.0 |
| haplotype 6 | 0.0 |
| haplotype 7 | 0.0 |
| haplotype 8 | 0.0 |
### Chart
| Category | Sales |
|---|---|
| haplotype 1 | 0.0 |
| haplotype 2 | 0.0 |
| haplotype 3 | 0.0 |
| haplotype 4 | 1.0 |
| haplotype 5 | 0.0 |
| haplotype 6 | 0.0 |
| haplotype 7 | 0.0 |
| haplotype 8 | 0.0 |
| h24 | 1.0 |
| h25 | 1.0 |
| h26 | 3.0 |
| h10 | 1.0 |
### Chart
| Category | Sales |
|---|---|
| haplotype 1 | 9.0 |
| haplotype 2 | 0.0 |
| haplotype 3 | 0.0 |
| haplotype 4 | 0.0 |
| haplotype 5 | 0.0 |
| haplotype 6 | 0.0 |
| haplotype 7 | 0.0 |
| haplotype 8 | 0.0 |
| h13 | 5.0 |
| h29 | 2.0 |
| h47 | 1.0 |
### Chart
| Category | Sales |
|---|---|
| haplotype 1 | 4.0 |
| haplotype 2 | 0.0 |
| haplotype 3 | 0.0 |
| haplotype 4 | 0.0 |
| haplotype 5 | 0.0 |
| haplotype 6 | 0.0 |
| haplotype 7 | 0.0 |
| haplotype 8 | 0.0 |
| h34 | 1.0 |
| h35 | 1.0 |
| h26 | 1.0 |H1
H2
H3
H4
H5
H6
H7
H8
H9
H10
H11
H12
H13
H14
H15
H16
H17
H18
H22
H23
H24
H25
H26
H27
H28
H29
H30
### Chart
| Category | Sales |
|---|---|
| haplotype 1 | 2.0 |
| haplotype 2 | 0.0 |
| haplotype 3 | 0.0 |
| haplotype 4 | 3.0 |
| haplotype 5 | 0.0 |
| haplotype 6 | 0.0 |
| haplotype 7 | 0.0 |
| haplotype 8 | 0.0 |
| h44 | 2.0 |
| h46 | 1.0 |
| h43 | 1.0 |
| h45 | 1.0 |
### Chart
| Category | Sales |
|---|---|
| haplotype 1 | 1.0 |
| haplotype 2 | 0.0 |
| haplotype 3 | 0.0 |
| haplotype 4 | 2.0 |
| haplotype 5 | 0.0 |
| haplotype 6 | 0.0 |
| haplotype 7 | 0.0 |
| haplotype 8 | 0.0 |
| h31 | 1.0 |
### Chart
| Category | Sales |
|---|---|
| haplotype 1 | 1.0 |
| haplotype 2 | 0.0 |
| haplotype 3 | 0.0 |
| haplotype 4 | 0.0 |
| haplotype 5 | 0.0 |
| haplotype 6 | 0.0 |
| haplotype 7 | 0.0 |
| haplotype 8 | 0.0 |
### Chart
| Category | Sales |
|---|---|
| H1 | 4.0 |
| H2 | 0.0 |
| H3 | 0.0 |
| H4 | 0.0 |
| H5 | 0.0 |
| H6 | 0.0 |
| H7 | 0.0 |
| H8 | 0.0 |
| H52 | 1.0 |
| H53 | 1.0 |
### Chart
| Category | Sales |
|---|---|
| haplotype 1 | 1.0 |
| haplotype 2 | 0.0 |
| haplotype 3 | 0.0 |
| haplotype 4 | 0.0 |
| haplotype 5 | 1.0 |
| haplotype 6 | 0.0 |
| haplotype 7 | 0.0 |
| haplotype 8 | 0.0 |
### Chart
| Category | Sales |
|---|---|
| haplotype 1 | 4.0 |
| haplotype 2 | 0.0 |
| haplotype 3 | 0.0 |
| haplotype 4 | 1.0 |
| haplotype 5 | 0.0 |
| haplotype 6 | 0.0 |
| haplotype 7 | 0.0 |
| haplotype 8 | 0.0 |
| h18 | 3.0 |
### Chart
| Category | Sales |
|---|---|
| haplotype 1 | 1.0 |
| haplotype 2 | 0.0 |
| haplotype 3 | 0.0 |
| haplotype 4 | 0.0 |
| haplotype 5 | 0.0 |
| haplotype 6 | 0.0 |
| haplotype 7 | 0.0 |
| haplotype 8 | 0.0 |
### Chart
| Category | Sales |
|---|---|
| haplotype 1 | 0.0 |
| haplotype 2 | 0.0 |
| haplotype 3 | 0.0 |
| haplotype 4 | 1.0 |
| haplotype 5 | 0.0 |
| haplotype 6 | 0.0 |
| haplotype 7 | 0.0 |
| haplotype 8 | 0.0 |
| h33 | 1.0 |
### Chart
| Category | Sales |
|---|---|
| haplotype 1 | 0.0 |
| haplotype 2 | 0.0 |
| haplotype 3 | 0.0 |
| haplotype 4 | 0.0 |
| haplotype 5 | 0.0 |
| haplotype 6 | 0.0 |
| haplotype 7 | 0.0 |
| haplotype 8 | 1.0 |
| h50 | 1.0 |
### Chart
| Category | Sales |
|---|---|
| haplotype 1 | 1.0 |
| haplotype 2 | 0.0 |
| haplotype 3 | 0.0 |
| haplotype 4 | 0.0 |
| haplotype 5 | 0.0 |
| haplotype 6 | 0.0 |
| haplotype 7 | 0.0 |
| haplotype 8 | 0.0 |
### Chart
| Category | Sales |
|---|---|
| haplotype 1 | 1.0 |
| haplotype 2 | 0.0 |
| haplotype 3 | 0.0 |
| haplotype 4 | 0.0 |
| haplotype 5 | 0.0 |
| haplotype 6 | 0.0 |
| haplotype 7 | 0.0 |
| haplotype 8 | 0.0 |
### Chart
| Category | Sales |
|---|---|
| haplotype 1 | 1.0 |
| haplotype 2 | 0.0 |
| haplotype 3 | 0.0 |
| haplotype 4 | 0.0 |
| haplotype 5 | 0.0 |
| haplotype 6 | 0.0 |
| haplotype 7 | 0.0 |
| haplotype 8 | 0.0 |H31
H32
H33
H34
H35
H36
H37
H38
H39
H40
H41
H42
H43
H44
H45
H46
H47
H48
H49
H50
H51
H52
H53
### Chart
| Category | Sales |
|---|---|
| haplotype 1 | 0.0 |
| haplotype 2 | 0.0 |
| haplotype 3 | 0.0 |
| haplotype 4 | 2.0 |
| haplotype 5 | 0.0 |
| haplotype 6 | 0.0 |
| haplotype 7 | 0.0 |
| haplotype 8 | 0.0 |
| h51 | 1.0 |
| h48 | 0.0 |
### Chart
| Category | Sales |
|---|---|
| haplotype 1 | 1.0 |
| haplotype 2 | 0.0 |
| haplotype 3 | 0.0 |
| haplotype 4 | 0.0 |
| haplotype 5 | 0.0 |
| haplotype 6 | 0.0 |
| haplotype 7 | 0.0 |
| haplotype 8 | 0.0 |
| h51 | 1.0 |
| h48 | 1.0 |
### Chart
| Category | Sales |
|---|---|
| haplotype 1 | 0.0 |
| haplotype 2 | 0.0 |
| haplotype 3 | 0.0 |
| haplotype 4 | 0.0 |
| haplotype 5 | 0.0 |
| haplotype 6 | 0.0 |
| haplotype 7 | 0.0 |
| haplotype 8 | 0.0 |
| h23 | 1.0 |
| h27 | 1.0 |
### Chart
| Category | Sales |
|---|---|
| haplotype 1 | 1.0 |
| haplotype 2 | 0.0 |
| haplotype 3 | 0.0 |
| haplotype 4 | 2.0 |
| haplotype 5 | 0.0 |
| haplotype 6 | 0.0 |
| haplotype 7 | 0.0 |
| haplotype 8 | 0.0 |
| h31 | 1.0 |
### Chart
| Category | Sales |
|---|---|
| haplotype 1 | 1.0 |
| haplotype 2 | 0.0 |
| haplotype 3 | 0.0 |
| haplotype 4 | 0.0 |
| haplotype 5 | 0.0 |
| haplotype 6 | 0.0 |
| haplotype 7 | 0.0 |
| haplotype 8 | 0.0 |
### Chart
| Category | Sales |
|---|---|
| haplotype 1 | 0.0 |
| haplotype 2 | 0.0 |
| haplotype 3 | 0.0 |
| haplotype 4 | 0.0 |
| haplotype 5 | 0.0 |
| haplotype 6 | 0.0 |
| haplotype 7 | 0.0 |
| haplotype 8 | 0.0 |
| h21 | 1.0 |
| h22 | 1.0 |
### Chart
| Category | Sales |
|---|---|
| haplotype 2 | 1.0 |
| haplotype 3 | 1.0 |
| haplotype 4 | 1.0 |
| haplotype 5 | 1.0 |
| haplotype 6 | 1.0 |
| haplotype 7 | 1.0 |
### Chart
| Category | Sales |
|---|---|
| haplotype 1 | 0.0 |
| haplotype 2 | 0.0 |
| haplotype 3 | 0.0 |
| haplotype 4 | 6.0 |
| haplotype 5 | 0.0 |
| haplotype 6 | 0.0 |
| haplotype 7 | 0.0 |
| haplotype 8 | 0.0 |
| h36 | 3.0 |
### Chart
| Category | Sales |
|---|---|
| haplotype 1 | 1.0 |
| haplotype 2 | 0.0 |
| haplotype 3 | 0.0 |
| haplotype 4 | 0.0 |
| haplotype 5 | 0.0 |
| haplotype 6 | 0.0 |
| haplotype 7 | 0.0 |
| haplotype 8 | 0.0 |
### Chart
| Category | Sales |
|---|---|
| haplotype 1 | 1.0 |
| haplotype 4 | 1.0 |
| h12 | 1.0 |
| h37 | 1.0 |
| h38 | 1.0 |
### Chart
| Category | Sales |
|---|---|
| haplotype 1 | 1.0 |
| haplotype 2 | 0.0 |
| haplotype 3 | 0.0 |
| haplotype 4 | 0.0 |
| haplotype 5 | 0.0 |
| haplotype 6 | 0.0 |
| haplotype 7 | 0.0 |
| haplotype 8 | 0.0 |
### Chart
| Category | Sales |
|---|---|
| haplotype 1 | 4.0 |
| haplotype 2 | 0.0 |
| haplotype 3 | 1.0 |
| haplotype 4 | 0.0 |
| haplotype 5 | 0.0 |
| haplotype 6 | 0.0 |
| haplotype 7 | 0.0 |
| haplotype 8 | 0.0 |
| h13 | 1.0 |
| h30 | 1.0 |
### Chart
| Category | Sales |
|---|---|
| H1 | 14.0 |
| H4 | 27.0 |
| H9 | 1.0 |
| H12 | 2.0 |
| H14 | 1.0 |
| H15 | 1.0 |
| H16 | 1.0 |
| H10 | 1.0 |
| H11 | 1.0 |
| H19 | 1.0 |
### Chart
| Category | Sales |
|---|---|
| haplotype 1 | 0.0 |
| haplotype 2 | 0.0 |
| haplotype 3 | 0.0 |
| haplotype 4 | 1.0 |
| haplotype 5 | 0.0 |
| haplotype 6 | 0.0 |
| haplotype 7 | 0.0 |
| haplotype 8 | 0.0 |
| h31 | 1.0 |
### Chart
| Category | Sales |
|---|---|
| haplotype 1 | 1.0 |
| haplotype 2 | 0.0 |
| haplotype 3 | 0.0 |
| haplotype 4 | 2.0 |
| haplotype 5 | 0.0 |
| haplotype 6 | 0.0 |
| haplotype 7 | 0.0 |
| haplotype 8 | 0.0 |
### Chart
| Category | Sales |
|---|---|
| haplotype 1 | 1.0 |
| haplotype 2 | 0.0 |
| haplotype 3 | 0.0 |
| haplotype 4 | 2.0 |
| haplotype 5 | 0.0 |
| haplotype 6 | 0.0 |
| haplotype 7 | 0.0 |
| haplotype 8 | 0.0 |
| h12 | 2.0 |
| h33 | 1.0 |
| h40 | 1.0 |
| h41 | 1.0 |
| h42 | 1.0 |
### Chart
| Category | Sales |
|---|---|
| haplotype 1 | 0.0 |
| haplotype 2 | 0.0 |
| haplotype 3 | 0.0 |
| haplotype 4 | 0.0 |
| haplotype 5 | 0.0 |
| haplotype 6 | 0.0 |
| haplotype 7 | 1.0 |
| haplotype 8 | 0.0 |
### Chart
| Category | Sales |
|---|---|
| haplotype 1 | 1.0 |
| haplotype 2 | 0.0 |
| haplotype 3 | 0.0 |
| haplotype 4 | 0.0 |
| haplotype 5 | 0.0 |
| haplotype 6 | 0.0 |
| haplotype 7 | 0.0 |
| haplotype 8 | 0.0 |
### Chart
| Category | Sales |
|---|---|
| haplotype 1 | 1.0 |
| haplotype 2 | 0.0 |
| haplotype 3 | 0.0 |
| haplotype 4 | 0.0 |
| haplotype 5 | 0.0 |
| haplotype 6 | 0.0 |
| haplotype 7 | 0.0 |
| haplotype 8 | 0.0 |H19
H20
H21
### Chart
| Category | Sales |
|---|---|
| haplotype 1 | 1.0 |
| haplotype 2 | 0.0 |
| haplotype 3 | 0.0 |
| haplotype 4 | 0.0 |
| haplotype 5 | 0.0 |
| haplotype 6 | 0.0 |
| haplotype 7 | 0.0 |
| haplotype 8 | 0.0 |
