# Supplementary material for: Homogeneity and Possible Replacement of Populations of the Dengue Vectors Aedes aegypti and Aedes albopictus in Indonesia
Source: Front Cell Infect Microbiol. 2021 Jul 7;11:705129. doi: 10.3389/fcimb.2021.705129 (PMC8294392; doi:10.3389/fcimb.2021.705129)
Supplement: Supplementary Figure 1 — cox1 gene phylogeny of the collected samples. The phylogenetic trees were built using maximum-likelihood (ML) with the general time reversible model with gama distributed with four discrete categories (GTR + I + G). The clade support was assessed via 500 bootstrap replicates. The tree was rooted using the Culex quinquefasciatus cox1 gene (MK265737) as outgroup. [file DataSheet_1.zip › Presentation 5.PPTX]

## Slide 1
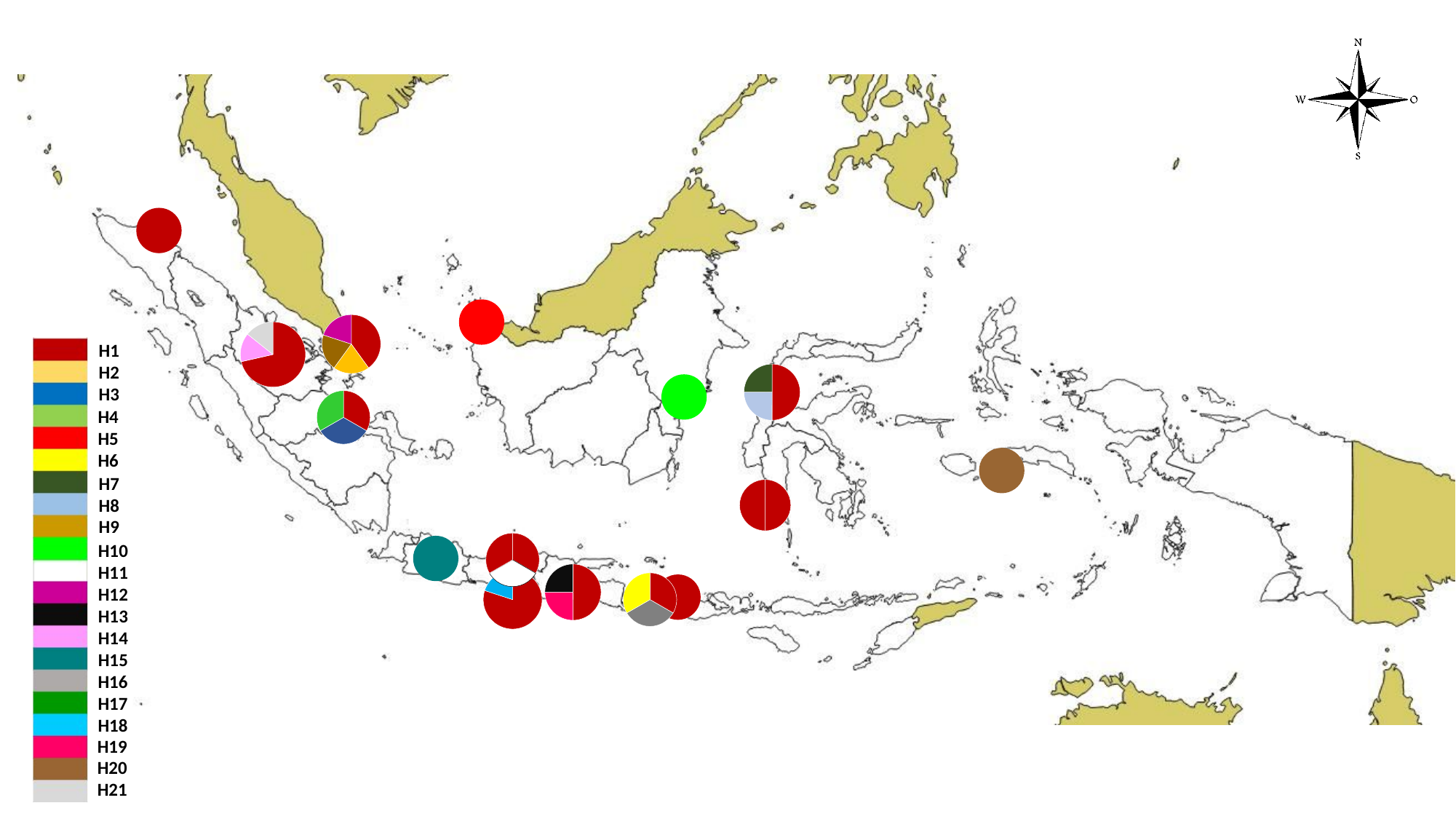

### Chart
| Category | Sales |
|---|---|
| haplotype 1 | 1.0 |
| haplotype 2 | 0.0 |
| haplotype 3 | 0.0 |
| haplotype 4 | 0.0 |
| haplotype 5 | 0.0 |
| haplotype 6 | 0.0 |
| haplotype 7 | 0.0 |
| haplotype 8 | 0.0 |
### Chart
| Category | Sales |
|---|---|
| haplotype 1 | 1.0 |
| haplotype 2 | 0.0 |
| haplotype 3 | 0.0 |
| haplotype 4 | 0.0 |
| haplotype 5 | 0.0 |
| haplotype 6 | 0.0 |
| haplotype 7 | 0.0 |
| haplotype 8 | 0.0 |
### Chart
| Category | Sales |
|---|---|
| haplotype 1 | 2.0 |
| haplotype 2 | 1.0 |
| haplotype 3 | 0.0 |
| haplotype 4 | 0.0 |
| haplotype 5 | 0.0 |
| haplotype 6 | 0.0 |
| haplotype 7 | 0.0 |
| haplotype 8 | 0.0 |
| H9 | 1.0 |
| H12 | 1.0 |
### Chart
| Category | Sales |
|---|---|
| haplotype 1 | 5.0 |
| haplotype 2 | 0.0 |
| haplotype 3 | 0.0 |
| haplotype 4 | 0.0 |
| haplotype 5 | 0.0 |
| haplotype 6 | 0.0 |
| haplotype 7 | 0.0 |
| haplotype 8 | 0.0 |
| H14 | 1.0 |
| H21 | 1.0 |
| h47 | 0.0 |H1
H2
H3
### Chart
| Category | Sales |
|---|---|
| haplotype 1 | 2.0 |
| haplotype 2 | 0.0 |
| haplotype 3 | 0.0 |
| haplotype 4 | 0.0 |
| haplotype 5 | 0.0 |
| haplotype 6 | 0.0 |
| haplotype 7 | 1.0 |
| haplotype 8 | 0.0 |
| h12 | 0.0 |
| h13 | 1.0 |
| h40 | 0.0 |
| h41 | 0.0 |
| h42 | 0.0 |
### Chart
| Category | Sales |
|---|---|
| haplotype 1 | 1.0 |
| haplotype 2 | 0.0 |
| haplotype 3 | 0.0 |
| haplotype 4 | 0.0 |
| haplotype 5 | 0.0 |
| haplotype 6 | 0.0 |
| haplotype 7 | 0.0 |
| haplotype 8 | 0.0 |
### Chart
| Category | Sales |
|---|---|
| haplotype 1 | 1.0 |
| haplotype 2 | 0.0 |
| haplotype 3 | 0.0 |
| haplotype 4 | 0.0 |
| haplotype 5 | 0.0 |
| haplotype 6 | 0.0 |
| haplotype 7 | 0.0 |
| haplotype 8 | 0.0 |
| h51 | 1.0 |
| h48 | 1.0 |H4
H5
H6
### Chart
| Category | Sales |
|---|---|
| haplotype 1 | 1.0 |
| haplotype 2 | 0.0 |
| haplotype 3 | 0.0 |
| haplotype 4 | 0.0 |
| haplotype 5 | 0.0 |
| haplotype 6 | 0.0 |
| haplotype 7 | 0.0 |
| haplotype 8 | 0.0 |H7
H8
H9
### Chart
| Category | Sales |
|---|---|
| haplotype 1 | 1.0 |
| haplotype 2 | 0.0 |
| haplotype 3 | 0.0 |
| haplotype 4 | 0.0 |
| haplotype 5 | 1.0 |
| haplotype 6 | 0.0 |
| haplotype 7 | 0.0 |
| haplotype 8 | 0.0 |
### Chart
| Category | Sales |
|---|---|
| haplotype 1 | 1.0 |
| haplotype 2 | 0.0 |
| haplotype 3 | 0.0 |
| haplotype 4 | 0.0 |
| haplotype 5 | 0.0 |
| haplotype 6 | 0.0 |
| haplotype 7 | 0.0 |
| haplotype 8 | 0.0 |
| h51 | 1.0 |
| h48 | 1.0 |H10
H11
H12
### Chart
| Category | Sales |
|---|---|
| haplotype 1 | 1.0 |
| haplotype 2 | 0.0 |
| haplotype 3 | 0.0 |
| haplotype 4 | 0.0 |
| haplotype 5 | 0.0 |
| haplotype 6 | 0.0 |
| haplotype 7 | 0.0 |
| haplotype 8 | 0.0 |
### Chart
| Category | Sales |
|---|---|
| haplotype 1 | 2.0 |
| haplotype 2 | 0.0 |
| haplotype 3 | 0.0 |
| haplotype 4 | 0.0 |
| haplotype 5 | 0.0 |
| haplotype 6 | 0.0 |
| haplotype 7 | 1.0 |
| haplotype 8 | 0.0 |
| h12 | 0.0 |
| h13 | 1.0 |
| h40 | 0.0 |
| h41 | 0.0 |
| h42 | 0.0 |
### Chart
| Category | Sales |
|---|---|
| haplotype 1 | 4.0 |
| haplotype 2 | 0.0 |
| haplotype 3 | 0.0 |
| haplotype 4 | 0.0 |
| haplotype 5 | 0.0 |
| haplotype 6 | 0.0 |
| haplotype 7 | 0.0 |
| haplotype 8 | 0.0 |
| H18 | 1.0 |
| H12 | 0.0 |
### Chart
| Category | Sales |
|---|---|
| haplotype 1 | 1.0 |
| haplotype 2 | 0.0 |
| haplotype 3 | 0.0 |
| haplotype 4 | 0.0 |
| haplotype 5 | 0.0 |
| haplotype 6 | 0.0 |
| haplotype 7 | 0.0 |
| haplotype 8 | 0.0 |
| h51 | 1.0 |
| h48 | 1.0 |
### Chart
| Category | Sales |
|---|---|
| haplotype 1 | 1.0 |
| haplotype 2 | 0.0 |
| haplotype 3 | 0.0 |
| haplotype 4 | 0.0 |
| haplotype 5 | 0.0 |
| haplotype 6 | 0.0 |
| haplotype 7 | 0.0 |
| haplotype 8 | 0.0 |H13
H14
H15
H16
H17
H18
H19
H20
H21
