# Supplementary figures and images for: Homogeneity and Possible Replacement of Populations of the Dengue Vectors Aedes aegypti and Aedes albopictus in Indonesia
Source: Front Cell Infect Microbiol. 2021 Jul 7;11:705129. doi: 10.3389/fcimb.2021.705129 (PMC8294392; doi:10.3389/fcimb.2021.705129)

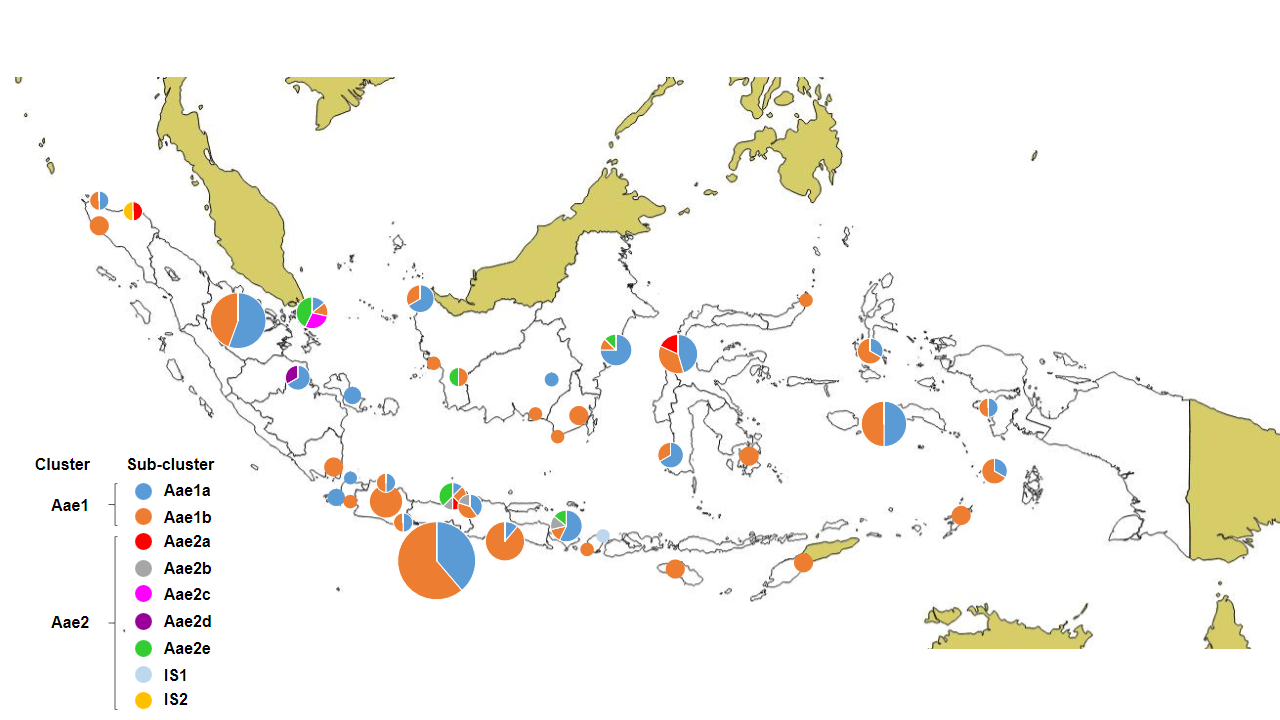

Supplement: Supplementary Figure 1 — cox1 gene phylogeny of the collected samples. The phylogenetic trees were built using maximum-likelihood (ML) with the general time reversible model with gama distributed with four discrete categories (GTR + I + G). The clade support was assessed via 500 bootstrap replicates. The tree was rooted using the Culex quinquefasciatus cox1 gene (MK265737) as outgroup. [file DataSheet_1.zip › Image 3.tif]

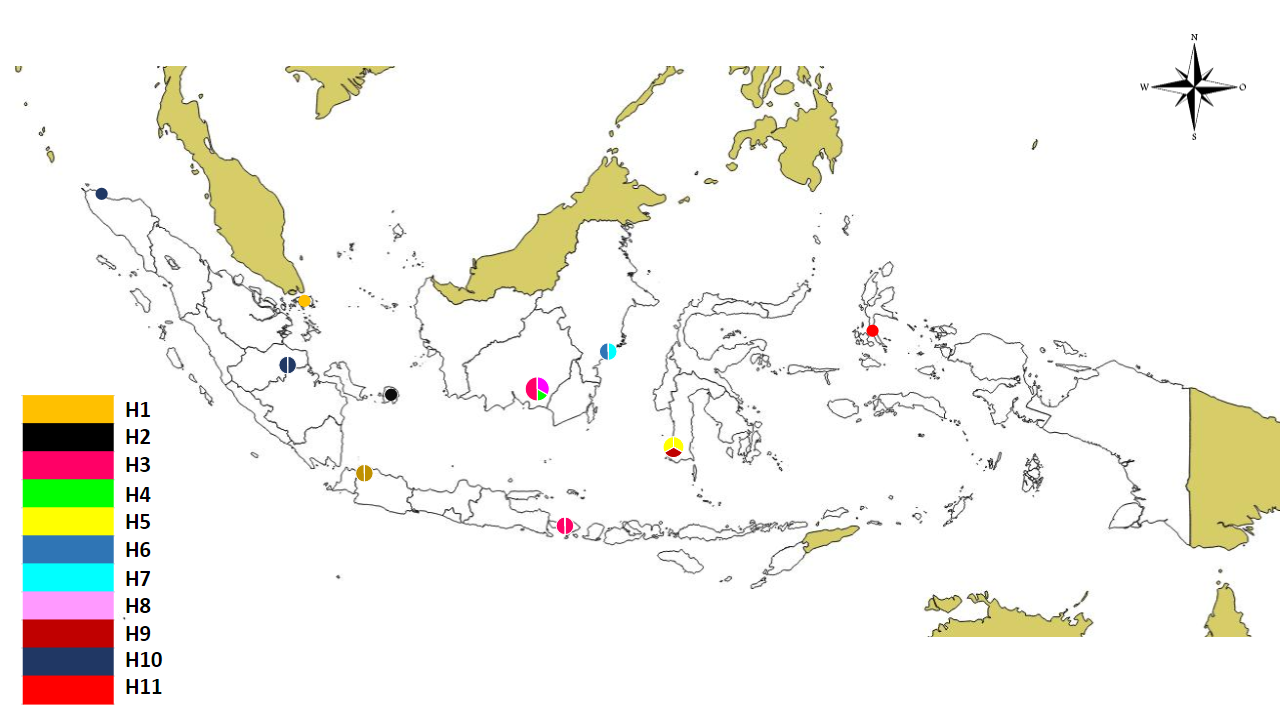

Supplement: Supplementary Figure 1 — cox1 gene phylogeny of the collected samples. The phylogenetic trees were built using maximum-likelihood (ML) with the general time reversible model with gama distributed with four discrete categories (GTR + I + G). The clade support was assessed via 500 bootstrap replicates. The tree was rooted using the Culex quinquefasciatus cox1 gene (MK265737) as outgroup. [file DataSheet_1.zip › Image 6.tif]

## Slide 1
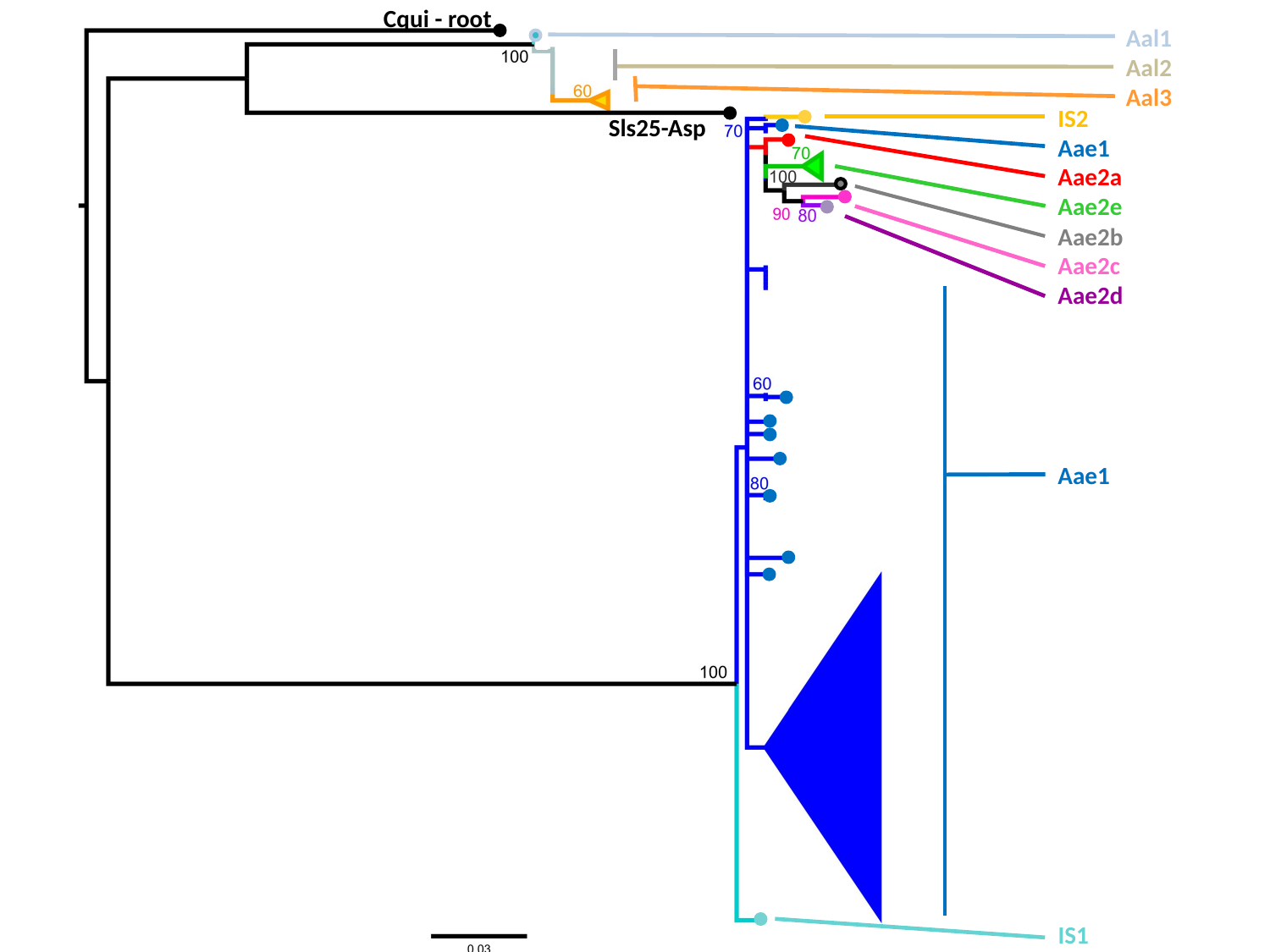

Cqui - root
Aal1
Aal2
Aal3
IS2
Aae1
Aae2a
Aae2e
Aae2b
Aae2c
Aae2d
Sls25-Asp
Aae1
IS1

Supplement: Supplementary Figure 1 — cox1 gene phylogeny of the collected samples. The phylogenetic trees were built using maximum-likelihood (ML) with the general time reversible model with gama distributed with four discrete categories (GTR + I + G). The clade support was assessed via 500 bootstrap replicates. The tree was rooted using the Culex quinquefasciatus cox1 gene (MK265737) as outgroup. [file DataSheet_1.zip › Presentation 1.PPTX]

## Slide 1
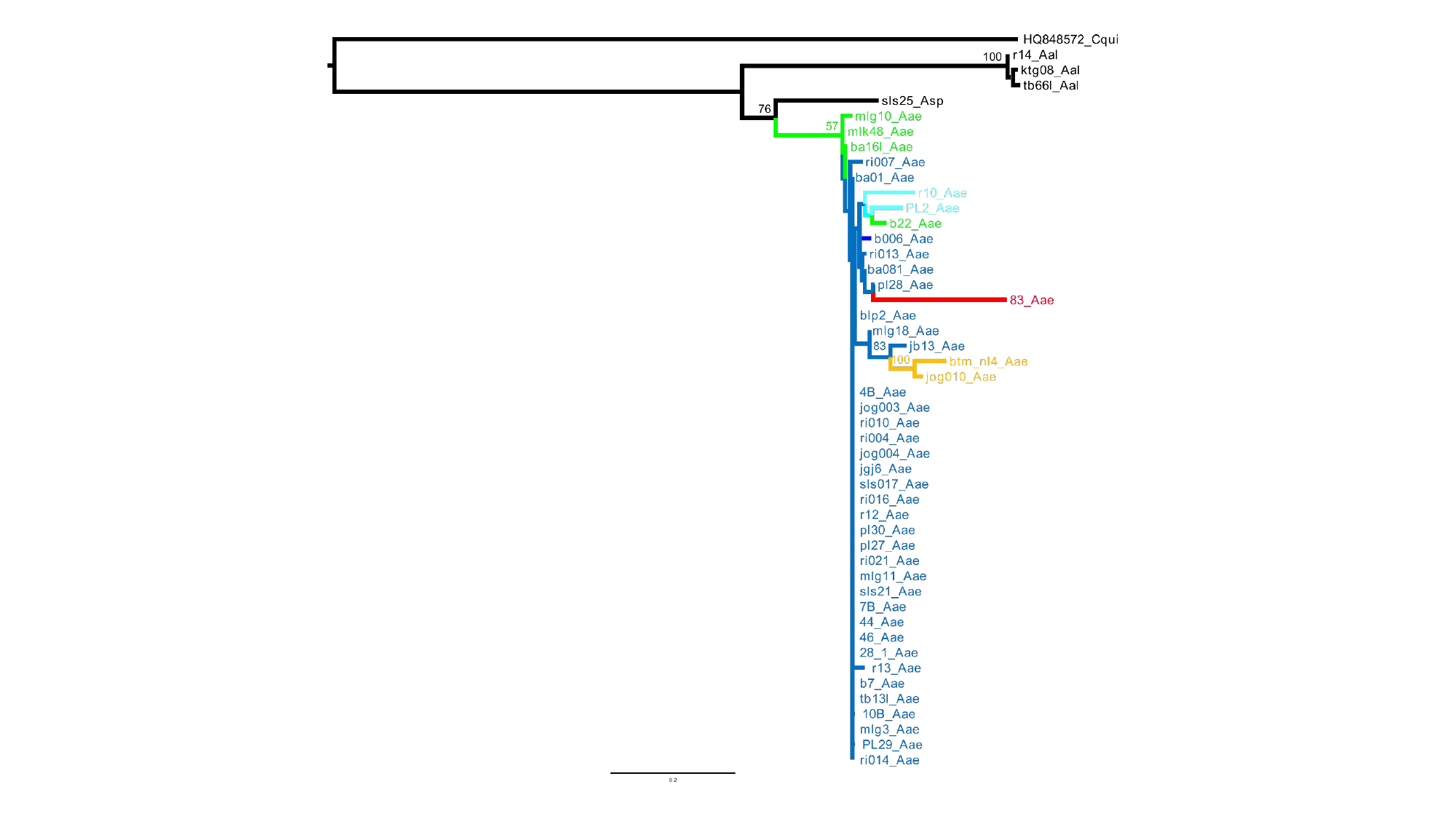

Supplement: Supplementary Figure 1 — cox1 gene phylogeny of the collected samples. The phylogenetic trees were built using maximum-likelihood (ML) with the general time reversible model with gama distributed with four discrete categories (GTR + I + G). The clade support was assessed via 500 bootstrap replicates. The tree was rooted using the Culex quinquefasciatus cox1 gene (MK265737) as outgroup. [file DataSheet_1.zip › Presentation 2.PPTX]
