# Supplementary material for: Homogeneity and Possible Replacement of Populations of the Dengue Vectors Aedes aegypti and Aedes albopictus in Indonesia
Source: Front Cell Infect Microbiol. 2021 Jul 7;11:705129. doi: 10.3389/fcimb.2021.705129 (PMC8294392; doi:10.3389/fcimb.2021.705129)
Supplement: Supplementary Table 1 — Specimens and sampling localities of Aedes aegypti. [file Table_1.pdf]

cox 1 Cluster	*cox* 1 Subcluster	*cox* 1 Haplotype	ITS2 Cluster	ITS2 Haplotype	Sample	Species	Location		*cox* 1 accession number	ITS2 accession number
							District/municipality	Province		
		H1			13_Aae	*Ae. aegypti*	Serang	Banten	MW280620	
		H1			57_Aae	*Ae. aegypti*	Bantul	Yogyakarta	MW280631	
		H1			4_Aae	*Ae. aegypti*	Bantul	Yogyakarta	MW280639	
		H1	1a	H1	r12_Aae	*Ae. aegypti*	Pematang Raman	Jambi	MW280645	MW290457
		H1			r011a_Aae	*Ae. aegypti*	Pematang Raman	Jambi	MW280646	
		H1	1a	H21	ri007_Aae	*Ae. aegypti*	Pekanbaru	Riau	MW280647	MW290466
		H1			ri017_Aae	*Ae. aegypti*	Pekanbaru	Riau	MW280661	
		H1			71_Aae	*Ae. aegypti*	Bantul	Yogyakarta	MW280663	
		H1			79_Aae	*Ae. aegypti*	Sambas	West Kalimantan	MW280666	
		H1			97_Aae	*Ae. aegypti*	Sambas	West Kalimantan	MW280667	
		H1	1b	H5	83_Aae	*Ae. aegypti*	Sambas	West Kalimantan	MW280673	MW322796
		H1			80_Aae	*Ae. aegypti*	Sambas	West Kalimantan	MW280674	
		H1	1a	H1	ri021_Aae	*Ae. aegypti*	Pekanbaru	Riau	MW280678	MW290454
		H1			20_Aae	*Ae. aegypti*	Pangandaran	West Java	MW280683	
		H1			mlk36_Aae	*Ae. aegypti*	Ambon	Maluku	MW280686	
		H1			mlk65_Aae	*Ae. aegypti*	Ambon	Maluku	MW280687	
		H1			mlk79_Aae	*Ae. aegypti*	Ambon	Maluku	MW280688	
		H1			blp28_Aae	*Ae. aegypti*	Balikpapan	East Kalimantan	MW280698	
		H1	1a	H14	ri013_Aae	*Ae. aegypti*	Pekanbaru	Riau	MW280700	MW290446
		H1			31_Aae	*Ae. aegypti*	Pidie	NAD	MW280703	
		H1			57_1_Aae	*Ae. aegypti*	Central Bangka	Bangka Belitung	MW280708	
		H1	2	H16	b22_Aae	*Ae. aegypti*	Karangasem	Bali	MW280710	MW290455
		H1	1a	H1	b7_Aae	*Ae. aegypti*	Karangasem	Bali	MW280713	MW290449
		H1			jgj5_Aae	*Ae. aegypti*	Bantul	Yogyakarta	MW280716	
		H1	1a	H1	jgj6_Aae	*Ae. aegypti*	Bantul	Yogyakarta	MW280717	MW290459
		H1			blp1b_Aae	*Ae. aegypti*	Balikpapan	East Kalimantan	MW280724	
		H1			jtg2b_Aae	*Ae. aegypti*	Semarang	Central Java	MW280739	
		H1	1a	H1	mlg11_Aae	*Ae. aegypti*	Malang	East Java	MW280743	MW290453
		H1	1a	H1	PL30_Aae	*Ae. aegypti*	Palu	Central Sulawesi	MW280755	MW290468
		H1			ri006b_Aae	*Ae. aegypti*	Pekanbaru	Riau	MW280759	
		H1			ri007_1_Aae	*Ae. aegypti*	Pekanbaru	Riau	MW280760	
		H1			ri021_1_Aae	*Ae. aegypti*	Pekanbaru	Riau	MW280762	
		H1	1	H1	sls21_Aae	*Ae. aegypti*	Maros	South Sulawesi	MW280763	MW290448
		H1			JOG11_Aae	*Ae. aegypti*	Bantul	Yogyakarta	MW280765	
		H1			58_Aae	*Ae. aegypti*	Central Bangka	Bangka Belitung	MW280769	
		H1			b14_Aae	*Ae. aegypti*	Karangasem	Bali	MW280770	
		H1			jgj7_Aae	*Ae. aegypti*	Bantul	Yogyakarta	MW280771	
	Aae1a	H1			blp15_1_Aae	*Ae. aegypti*	Balikpapan	East Kalimantan	MW280773	

		H1			r11_Aae	*Ae. aegypti*	Pematang Raman	Jambi	MW280778	
		H1			blp-3_Aae	*Ae. aegypti*	Balikpapan	East Kalimantan	MW280779	
		H1			8_1_Aae	*Ae. aegypti*	Southeast Maluku	Maluku	MW280784	
		H1			11_1_Aae	*Ae. aegypti*	Bantul	Yogyakarta	MW280786	
		H1			mlk40_Aae	*Ae. aegypti*	Ambon	Maluku	MW280790	
		H1			pl126_Aae	*Ae. Aegypti*	Palu	Central Sulawesi	MW280796	
		H1			19_1_Aae	*Ae. aegypti*	South Halmahera	North Maluku	MW280800	
		H1			blp11_Aae	*Ae. aegypti*	Balikpapan	East Kalimantan	MW280801	
		H1			ri002_Aae	*Ae. aegypti*	Pekanbaru	Riau	MW280802	
		H1			ri003_Aae	*Ae. aegypti*	Pekanbaru	Riau	MW280803	
		H1	1a	H1	ri013_1_Aae	*Ae. aegypti*	Pekanbaru	Riau	MW280805	
		H1			r12_1_Aae	*Ae. aegypti*	Pematang Raman	Jambi	MW280807	
		H1			jgj1_Aae	*Ae. aegypti*	Bantul	Yogyakarta	MW280809	
		H1			jgj2_Aae	*Ae. aegypti*	Bantul	Yogyakarta	MW280810	
		H1	1a	H1	jog003_Aae	*Ae. aegypti*	Bantul	Yogyakarta	MW280812	MW290451
		H1	1a	H1	jog004_Aae	*Ae. aegypti*	Bantul	Yogyakarta	MW280813	MW290460
		H1			jog006_Aae	*Ae. aegypti*	Bantul	Yogyakarta	MW280814	
		H1	1c	H18	jog010_Aae	*Ae. aegypti*	Bantul	Yogyakarta	MW280815	MW290461
		H1			b4_Aae	*Ae. aegypti*	Karangasem	Bali	MW280712	
		H2			1B_Aae	*Ae. aegypti*	Semarang City	Central Java	MW280621	
		H8			9_18_Aae	*Ae. aegypti*	Fak-Fak	West Papua	MW280628	
		H10			2_Aae	*Ae. aegypti*	Bantul	Yogyakarta	MW280637	
		H10	1c	H12	btm_nl4_Aae	*Ae. aegypti*	Batam	Riau Islands	MW280693	MW290444
		H11			6_Aae	*Ae. aegypti*	Bantul	Yogyakarta	MW280640	
		H14			44_Aae	*Ae. aegypti*	Bantul	Yogyakarta	MW280651	MW288143
		H15			27_Aae	*Ae. aegypti*	Bantul	Yogyakarta	MW280654	
		H16			28_Aae	*Ae. aegypti*	Bantul	Yogyakarta	MW280655	
		H20			15_1_Aae	*Ae. aegypti*	Pandeglang	Banten	MW280680	
		H20			14_Aae	*Ae. aegypti*	Pandeglang	Banten	MW280679	
		H22			19_Aae	*Ae. aegypti*	Subang	West Java	MW280682	
		H32			sk2_Aae	*Ae. aegypti*	Barito Kuala	South Kalimantan	MW280718	
		H34			blp15_Aae	*Ae. aegypti*	Balikpapan	East Kalimantan	MW280723	
		H37	1a	H1	JTG228_Aae	*Ae. aegypti*	Semarang	Central Java	MW280737	
		H44			PL27_Aae	*Ae. aegypti*	Palu	Central Sulawesi	MW280752	MW288144
		H44			PL5_Aae	*Ae. aegypti*	Palu	Central Sulawesi	MW280757	
		H46			PL4_Aae	*Ae. aegypti*	Palu	Central Sulawesi	MW280756	
		H48			sls17_Aae	*Ae. aegypti*	Maros	South Sulawesi	MW280764	MW288145
		H4			6B_Aae	*Ae. aegypti*	Semarang City	Central Java	MW280623	
		H4			5_Aae	*Ae. aegypti*	Bantul	Yogyakarta	MW280627	
		H4			25_Aae	*Ae. aegypti*	Bantul	Yogyakarta	MW280629	
		H4			47_Aae	*Ae. aegypti*	Bantul	Yogyakarta	MW280630	

Aae1		H4			23_Aae	*Ae. aegypti*	Bantul	Yogyakarta	MW280632	
		H4			38_Aae	*Ae. aegypti*	Bantul	Yogyakarta	MW280633	
		H4			52_Aae	*Ae. aegypti*	Bantul	Yogyakarta	MW280635	
		H4			1_Aae	*Ae. aegypti*	Bantul	Yogyakarta	MW280636	
		H4			3_Aae	*Ae. aegypti*	Bantul	Yogyakarta	MW280638	
		H4			7_Aae	*Ae. aegypti*	Bantul	Yogyakarta	MW280641	
		H4			24_Aae	*Ae. aegypti*	Bantul	Yogyakarta	MW280644	
		H4			50_Aae	*Ae. aegypti*	Bantul	Yogyakarta	MW280649	
		H4			41_Aae	*Ae. aegypti*	Bantul	Yogyakarta	MW280650	
		H4			15_Aae	*Ae. aegypti*	Bantul	Yogyakarta	MW280652	
		H4			26_Aae	*Ae. aegypti*	Bantul	Yogyakarta	MW280653	
		H4			55_Aae	*Ae. aegypti*	Bantul	Yogyakarta	MW280656	
		H4			60_Aae	*Ae. aegypti*	Bantul	Yogyakarta	MW280657	
		H4			63_Aae	*Ae. aegypti*	Bantul	Yogyakarta	MW280658	
		H4			69_Aae	*Ae. aegypti*	Bantul	Yogyakarta	MW280659	
		H4			76_Aae	*Ae. aegypti*	Bantul	Yogyakarta	MW280660	
		H4			53_Aae	*Ae. aegypti*	Bantul	Yogyakarta	MW280662	
		H4			75_Aae	*Ae. aegypti*	Bantul	Yogyakarta	MW280664	
		H4			67_Aae	*Ae. aegypti*	Bantul	Yogyakarta	MW280665	
		H4			62_Aae	*Ae. aegypti*	Bantul	Yogyakarta	MW280668	
		H4			68_Aae	*Ae. aegypti*	Bantul	Yogyakarta	MW280669	
		H4			74_Aae	*Ae. aegypti*	Bantul	Yogyakarta	MW280671	
		H4			pl1_Aae	*Ae. aegypti*	Palu	Central Sulawesi	MW280676	
		H4			21_Aae	*Ae. aegypti*	Pangandaran	West Java	MW280684	
		H4			25_1_Aae	*Ae. aegypti*	Bitung	North Sulawesi	MW280689	
		H4	1a	H9	ba081_Aae	*Ae. aegypti*	Batam	Riau Islands	MW280691	MW290438
		H4			pl1_1_Aae	*Ae. aegypti*	Palu	Central Sulawesi	MW280699	
		H4			32_Aae	*Ae. aegypti*	West Aceh	NAD	MW280704	
		H4			33_Aae	*Ae. aegypti*	West Aceh	NAD	MW280705	
		H4			47_1_Aae	*Ae. aegypti*	West Lombok	West Nusa Tenggara	MW280706	
		H4			52_1_Aae	*Ae. aegypti*	North kayong	West Kalimantan	MW280707	
		H4			sk5_Aae	*Ae. aegypti*	Kota Baru	South Kalimantan	MW280720	
		H4			sk6_Aae	*Ae. aegypti*	Tanah laut	South Kalimantan	MW280721	
		H4			sk56_Aae	*Ae. aegypti*	Ketapang	West Kalimantan	MW280722	
		H4			jb10_Aae	*Ae. aegypti*	West Bandung	West Java	MW280727	
		H4	1a	H15	jb13_Aae	*Ae. aegypti*	West Bandung	West Java	MW280729	MW290450
		H4			jb14_Aae	*Ae. aegypti*	West Bandung	West Java	MW280730	
		H4			jb15_Aae	*Ae. aegypti*	West Bandung	West Java	MW280731	
		H4			jb16_Aae	*Ae. aegypti*	West Bandung	West Java	MW280732	
		H4			jb19_Aae	*Ae. aegypti*	West Bandung	West Java	MW280735	
		H4			JTG10_Aae	*Ae. aegypti*	Semarang	Central Java	MW280736	

	Aae1b	H4	1a	H19	mlg18_Aae	*Ae. aegypti*	Malang	East Java	MW280745	MW290462
		H4	1a	H17	PL28_Aae	*Ae. aegypti*	Palu	Central Sulawesi	MW280753	MW290456
		H4			43_Aae	*Ae. aegypti*	Belu	East Nusa Tenggara	MW280767	
		H4			38_1_Aae	*Ae. aegypti*	Central Sumba	East Nusa Tenggara	MW280774	
		H4			43_1_Aae	*Ae. aegypti*	Belu	East Nusa Tenggara	MW280776	
		H4			3_1_Aae	*Ae. aegypti*	Southeast Maluku	Maluku	MW280782	
		H4			6_1_Aae	*Ae. aegypti*	Southeast Maluku	Maluku	MW280783	
		H4			10_1_Aae	*Ae. aegypti*	Bantul	Yogyakarta	MW280785	
		H4			13_11_1_Aae	*Ae. aegypti*	South Halmahera	North Maluku	MW280787	
		H4			15_1_1_Aae	*Ae. aegypti*	Muna	Southeast Sulawesi	MW280788	
		H4			mlg9_Aae	*Ae. aegypti*	Malang	East Java	MW280750	
		H4			mlk73_Aae	*Ae. aegypti*	Ambon	Maluku	MW280792	
		H4			pl1_1_1_Aae	*Ae. aegypti*	Palu	Central Sulawesi	MW280795	
		H4			3843_Aae	*Ae. aegypti*	Central Sumba	East Nusa Tenggara	MW280816	
		H4			37_Aae	*Ae. aegypti*	West Southeast Maluku	Maluku	MW280817	
		H6			8B_Aae	*Ae. aegypti*	Semarang City	Central Java	MW280625	
		H9			jgj8_Aae	*Ae. aegypti*	Bantul	Yogyakarta	MW280634	
		H12			JTG27_Aae	*Ae. aegypti*	Semarang	Central Java	MW280738	
		H12			mlg12_Aae	*Ae. aegypti*	Malang	East Java	MW280744	
		H12			mlg21_Aae	*Ae. aegypti*	Malang	East Java	MW280748	
		H12			8_Aae	*Ae. aegypti*	Bantul	Yogyakarta	MW280642	
		H12			jgj3_Aae	*Ae. aegypti*	Bantul	Yogyakarta	MW280811	
		H13			ri011_Aae	*Ae. aegypti*	Pekanbaru	Riau	MW280804	
		H13			ri024_Aae	*Ae. aegypti*	Pekanbaru	Riau	MW280806	
		H13	1a	H1	ri004_Aae	*Ae. aegypti*	Pekanbaru	Riau	MW280643	MW290463
		H13	1a	H1	ri016_Aae	*Ae. aegypti*	Pekanbaru	Riau	MW280648	MW290458
		H13	1a	H1	ri014_Aae	*Ae. aegypti*	Pekanbaru	Riau	MW280677	MW290447
		H13			ktg_H05_Aae	*Ae. aegypti*	Pulang Pisau	Central Kalimantan	MW280741	
		H13			b8_Aae	*Ae. aegypti*	Karangasem	Bali	MW280714	
		H17			82_Aae	*Ae. aegypti*	Sambas	West Kalimantan	MW280670	
		H18			81_Aae	*Ae. aegypti*	Sambas	West Kalimantan	MW280672	
		H18			2_1_Aae	*Ae. aegypti*	Lebak	Banten	MW280781	
		H18	2	H20	mlk48_Aae	*Ae. aegypti*	Ambon	Maluku	MW280791	MW290465
		H18			mlk654_Aae	*Ae. aegypti*	Ambon	Maluku	MW280794	
		H18			mlk54_Aae	*Ae. aegypti*	Ambon	Maluku	MW280772	
		H19			56_Aae	*Ae. aegypti*	Bantul	Yogyakarta	MW280675	
		H21			18_Aae	*Ae. aegypti*	Subang	West Java	MW280681	
		H23			66_Aae	*Ae. aegypti*	South Lampung	Lampung	MW280685	
		H27	1a	H1	65_Aae	*Ae. aegypti*	South Lampung	Lampung	MW280696	
		H29	1a	H1	ri010_Aae	*Ae. aegypti*	Pekanbaru	Riau	MW280701	MW290464
		H29			ri008_Aae	*Ae. aegypti*	Pekanbaru	Riau	MW280761	

		H31			15_18_1_Aae	*Ae. aegypti*	West Southeast Maluku	Maluku	MW280818	
		H31			15_18_Aae	*Ae. aegypti*	South Halmahera	North Maluku	MW280715	
		H33			sk4_Aae	*Ae. aegypti*	Kota Baru	South Kalimantan	MW280719	
		H35			blp23_Aae	*Ae. aegypti*	Balikpapan	East Kalimantan	MW280726	
		H36			jb11_Aae	*Ae. aegypti*	West Bandung	West Java	MW280728	
		H36			jb17_Aae	*Ae. aegypti*	West Bandung	West Java	MW280733	
		H36			jb18_Aae	*Ae. aegypti*	West Bandung	West Java	MW280734	
		H39	2	H13	mlg10_Aae	*Ae. aegypti*	Malang	East Java	MW280742	MW290445
		H40			mlg19_Aae	*Ae. aegypti*	Malang	East Java	MW280746	
		H41	1a	H1	mlg20_Aae	*Ae. aegypti*	Malang	East Java	MW280747	
		H42	1a	H1	mlg3_Aae	*Ae. aegypti*	Malang	East Java	MW280749	MW290452
		H47	1a	H1	ri004b_Aae	*Ae. aegypti*	Pekanbaru	Riau	MW280758	
		H50			9_18_1_Aae	*Ae. aegypti*	Fak-Fak	West Papua	MW280768	
		H51			sls32_Aae	*Ae. aegypti*	Maros	South Sulawesi	MW280775	
		H51			16_1_Aae	*Ae. aegypti*	Muna	Southeast Sulawesi	MW280789	
Aae2	Aae2a	H7	1a	H11	10B_Aae	*Ae. aegypti*	Semarang City	Central Java	MW280626	MW290443
	Aae2a	H7			29_Aae	*Ae. aegypti*	East Aceh	NAD	MW280702	
	Aae2a	H43	1d	H8	PL2_Aae	*Ae. aegypti*	Palu	Central Sulawesi	MW280751	MW290437
	Aae2a	H45	1a	H7	PL29_Aae	*Ae. aegypti*	Palu	Central Sulawesi	MW280754	MW290436
	Aae2b	H5	1a	H1	7B_Aae	*Ae. aegypti*	Semarang City	Central Java	MW280624	MW290442
	Aae2b	H30	1a	H6	b006_Aae	*Ae. aegypti*	Karangasem	Bali	MW280709	MW290435
	Aae2b	H38	1a	H1	jtg44_Aae	*Ae. aegypti*	Semarang	Central Java	MW280740	MW290467
	Aae2c	H24	1a	H1	ba01_Aae	*Ae. aegypti*	Batam	Riau Islands	MW280690	MW290433
	Aae2c	H25	2	H2	ba16l_Aae	*Ae. aegypti*	Batam	Riau Islands	MW280692	
	Aae2d	H52	1d	H4	r10_Aae	*Ae. aegypti*	Pematang Raman	Jambi	MW280777	
	Aae2d	H53	1a	H3	r13_Aae	*Ae. aegypti*	Pematang Raman	Jambi	MW280808	MW290434
	Aae2e	H3	1a	H1	4B_Aae	*Ae. aegypti*	Semarang City	Central Java	MW280622	MW290441
	Aae2e	H3			b3_Aae	*Ae. aegypti*	Karangasem	Bali	MW280622	
	Aae2e	H26	1a	H1	tb131_Aae	*Ae. aegypti*	Batam	Riau Islands	MW280797	MW290439
	Aae2e	H26			tb71l_Aae	*Ae. aegypti*	Batam	Riau Islands	MW280695	
	Aae2e	H26	1a	H10	blp2_Aae	*Ae. aegypti*	Balikpapan	East Kalimantan	MW280725	MW290440
	Aae2e	H26			55_1_Aae	*Ae. aegypti*	Ketapang	West Kalimantan	MW280780	
Individual samples	IS1	H49	1a	H1	46_Aae	*Ae. aegypti*	North Lombok	West Nusa Tenggara	MW280766	MW290432
	IS2	H28	1a	H1	28_1_Aae	*Ae. aegypti*	East Aceh	NAD	MW280697	MW290431

NAD: Nangro Aceh Darussalam (new denomination of the former Province of Aceh)
