# Supplementary material for: Homogeneity and Possible Replacement of Populations of the Dengue Vectors Aedes aegypti and Aedes albopictus in Indonesia
Source: Front Cell Infect Microbiol. 2021 Jul 7;11:705129. doi: 10.3389/fcimb.2021.705129 (PMC8294392; doi:10.3389/fcimb.2021.705129)
Supplement: Supplementary Table 2 — Specimens and sampling localities of Aedes albopictus. [file Table_2.pdf]

COI cluster	COI Haplotype	Sample	Species	Location	
				District/municipality	Province
Aal1	H2	61_Aal	*Ae. albopictus*	Belitung	Bangka Belitung
Aal1	H3	b023_Aal	*Ae. albopictus*	Denpasar	Bali
Aal1	H3	b24_Aal	*Ae. albopictus*	Denpasar	Bali
Aal1	H6	blp1_Aal	*Ae. albopictus*	Balikpapan	East Kalimantan
Aal1	H9	sls30_Aal	*Ae. albopictus*	Maros	South Sulawesi
Aal1	H5	sls95_Aal	*Ae. albopictus*	Maros	South Sulawesi
Aal1	H3	ktg06_Aal	*Ae. albopictus*	Pulang Pisau	Central Kalimantan
Aal1	H3	ktgp09_Aal	*Ae. albopictus*	Pulang Pisau	Central Kalimantan
Aal1	H4	ktgp26_Aal	*Ae. albopictus*	Pulang Pisau	Central Kalimantan
Aal1	H5	sls16_Aal	*Ae. albopictus*	Maros	South Sulawesi
Aal1	H7	blp20_Aal	*Ae. albopictus*	Balikpapan	East Kalimantan
Aal2	H8	ktg08_Aal	*Ae. albopictus*	Pulang Pisau	Central Kalimantan
Aal2	H8	ktgt14_Aal	*Ae. albopictus*	Pulang Pisau	Central Kalimantan
Aal2	H8	ktgp28	*Ae. albopictus*	Pulang Pisau	Central Kalimantan
Aal3	H10	r14_Aal	*Ae. albopictus*	Jambi	Jambi
Aal3	H10	30_Aal	*Ae. albopictus*	Pidie	Aceh
Aal3	H10	r15_Aal	*Ae. albopictus*	Jambi	Jambi
Aal3	H1	TB66l_Aal	*Ae. albopictus*	Batam	Riau Islands
Aal3	H11	22_30_Aal	*Ae. albopictus*	South Halmahera	North Maluku

cox 1 accession number	ITS2 accession number
MW283306	
MW283307	
MW283308	
MW283309	
MW283313	
MW283315	
MW280798	
MW280793	
MW283303	
MW283304	
MW283310	
MW283311	MW287155
MW283312	
MW280799	
MW283314	MW287156
MW283318	
MW283317	
MW283305	MW287157
MW283316	
