# Supplementary material for: Homogeneity and Possible Replacement of Populations of the Dengue Vectors Aedes aegypti and Aedes albopictus in Indonesia
Source: Front Cell Infect Microbiol. 2021 Jul 7;11:705129. doi: 10.3389/fcimb.2021.705129 (PMC8294392; doi:10.3389/fcimb.2021.705129)
Supplement: Supplementary Table 3 — Polymorphism of Aedes aegypti cox1 haplotypes from Indonesia. [file Table_3.docx]

**Supplementary Table 3. Polymorphism of *Aedes aegypti cox*1 haplotypes from Indonesia**

Cluster Cluster best hit ^a^ Location Haplotype % identity ^b^

1 MN299016 Peru H1 99.67 %

MN299014 Cambodia H2 99.18 %

MN299008 Puerto Rico H4 99.51 %

MG198586 Georgia H6 99.34 %

MF999266 India H8 99.18 %

MF043259 England H9 99.34 %

KY022527 Germany H10 99.34 %

KY022526 Germany H11 99.34 %

AF425846 NA H12 99.51 %

H13 99.51 %

H14 98.85 %

H15 99.34 %

H16 99.01 %

H17 99.34 %

H18 99.51 %

H19 99.34 %

H20 99.34 %

H21 99.34 %

H22 99.34 %

H23 99.34 %

H27 99.51 %

H29 99.51 %

H31 99.34 %

H32 99.34 %

H33 99.51 %

H34 99.34 %

H35 99.51 %

H36 99.51 %

H37 99.18 %

H39 99.51 %

H40 99.18 %

H41 99.34 %

H42 99.34 %

H44 99.51 %

H46 99.34 %

H47 99.51 %

H48 99.34 %

H50 99.18 %

H51 99.51 %

2a MK300222 Kenya H7 99.51 %

MK300216 Kenya H43 99.34 %

H45 99.18 %

2b MN299002 Mozambique H5 98.69 %

H30 98.69 %

H38 99.18 %

2c MT328866 Egypt H24 99.18 %

MK300229 Kenya H25 99.34 %

MK300226 Kenya

MK300223 Kenya

MK300217 Kenya

2d MN298993 Haiti H52 99.51 %

H53 99.67 %

2e MN298997 Haiti H3 99.67 %

AY432106 Strain Liverpool H26 99.84 %

AY432648 Strain Liverpool

AF390098 Strain RED

IS1 AY056597 Sub-Saharan Africa ^c^ H49 99.18 %

IS2 MH251910 Russia H28 98.85 %

a) All sequences displaying the same best hit score were reported with their respective accession number

b) The percentage of identity of a given haplotype is the same for each best hit sequence

c) The best hit corresponds to the form *Ae. aegypti formosus* which is considered to be the ancestral form of *Ae. aegypti*.
