# Supplementary material for: Homogeneity and Possible Replacement of Populations of the Dengue Vectors Aedes aegypti and Aedes albopictus in Indonesia
Source: Front Cell Infect Microbiol. 2021 Jul 7;11:705129. doi: 10.3389/fcimb.2021.705129 (PMC8294392; doi:10.3389/fcimb.2021.705129)
Supplement: Supplementary Table 4 — Polymorphism of Aedes albopictus cox1 haplotypes from Indonesia. [file Table_4.docx]

**Supplementary Table 4. Polymorphism of *Aedes albopictus cox*1 haplotypes from Indonesia**

Haplogroup Cluster best hit ^a^ Location Haplotype % identity ^b^

A2a KX809764 Philippines H1 99.67 %

KX809761 Philippines H2 99.84 %

KX383935 Philippines H3 99.51 %

H4 99.67 %

A1b1 MN299017 D. R. Congo H5 99.84%

KU738429 China H6 99.67 %

KU738428 China

KU738427 China

KU738426 China

KU738425 China

KU738424 China

KX383928 Thailand

KX383927 Greece

KX383926 Greece

KX383925 Thailand

KX383924 Brazil

KC690951 USA

KC690941 USA

KC690940 USA

a) All sequences displaying the same best hit score were reported with their respective accession number

b) The percentage of identity of a given haplotype is the same for each best hit sequence
