# Supplementary material for: Homogeneity and Possible Replacement of Populations of the Dengue Vectors Aedes aegypti and Aedes albopictus in Indonesia
Source: Front Cell Infect Microbiol. 2021 Jul 7;11:705129. doi: 10.3389/fcimb.2021.705129 (PMC8294392; doi:10.3389/fcimb.2021.705129)
Supplement: Supplementary Table 5 — Polymorphism of Aedes aegypti ITS2 haplotypes from Indonesia. [file Table_5.docx]

**Supplementary Table 5. Polymorphism of *Aedes aegypti* ITS2 haplotypes from Indonesia**

Cluster Cluster best hit ^a^ Location Haplotype % identity ^b^

1a MH142327 Russia H1 99.65 %

KY382418 Sri Lanka H2 99.30 %

HE820724 Russia H3 99.30 %

H6 98.25%

H7 98.25%

H8 98.23%

H4 97.55%

H5 97.54%

H9 97.20%

H10 94.68%

1b MH142327 Russia H11 99.51%

MH142320 Russia

MH142318 Russia

KY328418 Sri Lanka

KF471584 Rockefeller Strain

KF471587 Rockefeller Strain

KF471579 New Caledonia

(France)

KP259840 India

HE820724 Russia

1c KF471584 Rockefeller Strain H12 91.29%

MH142327 Russia 91.23%^c^

KY382418 Sri Lanka 91.23%^c^

HE820724 Russia 91.23%^c^

1d KF471584 Rockefeller strain H13 88.24%

MH142327 Russia 88.11%^c^

KY382418 Sri Lanka 88.11%^c^

HE820724 Russia 88.11%^c^

1e KF471579 New Caledonia H14 94.93 %

(France)

KF471584 Rockefeller Strain H15 92.61%

1f KU497614 NA H16 99.65%

KU497614 NA H17 99.65%

KU497614 NA H18 99.65%

KU497614 NA H19 96.14%

a) All sequences displaying the same best hit score were reported with their respective accession number

b) The percentage of identity of a given haplotype is the same for each best hit sequence

c) Second best hit score
