# Supplementary material for: Homogeneity and Possible Replacement of Populations of the Dengue Vectors Aedes aegypti and Aedes albopictus in Indonesia
Source: Front Cell Infect Microbiol. 2021 Jul 7;11:705129. doi: 10.3389/fcimb.2021.705129 (PMC8294392; doi:10.3389/fcimb.2021.705129)
Supplement: Supplementary Table 6 — Polymorphism of Aedes albopictus ITS2 haplotypes from Indonesia. [file Table_6.docx]

**Supplementary Table 6. Polymorphism of *Aedes albopictus* ITS2 haplotypes from Indonesia**

Sample Best hit ^a^ Location Haplotype % identity ^b^

ktg08_Aal MN062760 Israel H1 98.82 %

MN062758 Israel

MN062754 Israel

MN062753 Israel

MN062749 Israel

MN062743 Israel

MN062742 Israel

KY382421 Sri Lanka

KF471600 Italy

KF471594 Italy

KF471591 Italy

JX679394 Italy

JX679391 Italy

JX679390 Italy

JX679387 Italy

r14_Aal MH142323 Georgia H2 100 %

JX679389 Italy

TB66L_Aal MH142322 Georgia H3 99.41 %

KF471598 Italy

KF471595 Italy

JX679395 Italy

a) All sequences displaying the same best hit score were reported with their respective accession number

b) The percentage of identity of a given haplotype is the same for each best hit sequence
